# Single‐Component Dual‐Functional Autoboost Strategy by Dual Photodynamic and Cyclooxygenase‐2 Inhibition for Lung Cancer and Spinal Metastasis

**DOI:** 10.1002/advs.202303981

**Published:** 2024-01-15

**Authors:** Ben Wang, Zhen‐Ni Lu, Meng‐Xiong Song, Xiao‐Wen He, Zhi‐Chao Hu, Hai‐Feng Liang, Hong‐Wei Lu, Qing Chen, Bing Liang, Tao Yi, Peng Wei, Li‐Bo Jiang, Jian Dong

**Affiliations:** ^1^ Department of Orthopaedic Surgery Zhongshan Hospital Fudan University Shanghai 200032 China; ^2^ State Key Laboratory for Modification of Chemical Fibers and Polymer Materials College of Chemistry and Chemical Engineering Donghua University Shanghai 201620 China; ^3^ Department of Orthopedics Surgery Minhang Hospital Fudan University Shanghai 201100 China; ^4^ Department of Orthopaedic Surgery Shanghai Baoshan District Wusong Center Hospital Fudan University Shanghai 200940 China

**Keywords:** COX‐2, HOCl response, immune activation, photodynamic therapy

## Abstract

Coloading adjuvant drugs or biomacromolecules with photosensitizers into nanoparticles to enhance the efficiency of photodynamic therapy (PDT) is a common strategy. However, it is difficult to load positively charged photosensitizers and negatively charged adjuvants into the same nanomaterial and further regulate drug release simultaneously. Herein, a single‐component dual‐functional prodrug strategy is reported for tumor treatment specifically activated by tumor microenvironment (TME)‐generated HOCl. A representative prodrug (DHU‐CBA2) is constructed using indomethacin grafted with methylene blue (MB). DHU‐CBA2 exhibited high sensitivity toward HOCl and achieved simultaneous release of dual drugs in vitro and in vivo. DHU‐CBA2 shows effective antitumor activity against lung cancer and spinal metastases via PDT and cyclooxygenase‐2 (COX‐2) inhibition. Mechanistically, PDT induces immunogenic cell death but stimulates the gene encoding COX‐2. Downstream prostaglandins E_2_ and Indoleamine 2,3 dioxygenase 1 (IDO1) mediate immune escape in the TME, which is rescued by the simultaneous release of indomethacin. DHU‐CBA2 promotes infiltration and function of CD8^+^ T cells, thus inducing a robust antitumor immune response. This work provides an autoboost strategy for a single‐component dual‐functional prodrug activated by TME‐specific HOCl, thereby achieving favorable tumor treatment via the synergistic therapy of PDT and a COX‐2 inhibitor.

## Graphical abstract

This article presents a single‐component, dual‐functional prodrug, DHU‐CBA2, designed to simultaneously release indomethacin and methylene blue (MB) in response to HOCl. Both in vitro and in vivo studies demonstrate the high sensitivity of DHU‐CBA2 toward HOCl. The prodrug triggers a potent antitumor immune response by combining PDT and cyclooxygenase‐2 inhibition, and exhibits effective antitumor activity.

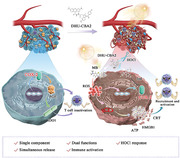

## 1 Introduction

Lung cancer (LC), the leading cause of global cancer‐related deaths, severely threatens personal health and causes an enormous economic burden.^[^ ^1^ ^]^ Conventional therapies, including surgery, chemotherapy, and radiotherapy, have inevitable systemic side effects, such as sequelae of anesthesia, hemorrhage, and systemic multi‐organ toxicity.^[^ ^2^ ^]^ Fortunately, photodynamic therapy (PDT) holds promise for reducing the side effects during cancer treatment.^[^ ^2^, ^3^ ^]^ This emerging cancer treatment converts oxygen (O_2_) into singlet oxygen (^1^O_2_) to kill cancer cells and has unique advantages, including noninvasiveness, excellent controllability, and minimal toxicity.^[^ ^4^ ^]^ However, previous studies have reported that the antitumor capacity of PDT alone is too limited to completely or highly efficiently ablate tumors. Various approaches have been used to combine PDT with other treatments, such as chemotherapy. However, these treatments aim to improve the effectiveness of chemotherapeutic drugs rather than fundamentally address the drawbacks of PDT. In addition to directly killing tumor tissues, some studies have reported that PDT exhibits a powerful function in immune activation, known as immune‐activating PDT (imPDT).^[^ ^5^ ^]^ However, with in‐depth studies, apparent drawbacks of PDT that adversely affect the immune microenvironment have been uncovered, which may severely impair its antitumor efficacy.^[^ ^5^, ^6^ ^]^ Thus, a more effective combination treatment using PDT for cancer should identify critical gene changes and related products induced in tumor cells capable of conferring immune escape in the tumor microenvironment (TME).

PDT significantly increases cyclooxygenase 2 (COX‐2) expression.^[^ ^5^, ^6^ ^]^ COX‐2, known as prostaglandin G/H synthase‐2, is typically low‐expressed in normal tissues but high‐expressed in many human tumors.^[^ ^7^ ^]^ It converts arachidonic acid to the endogenous peroxidation intermediate PGH2, which is further modified to prostaglandins via prostaglandin synthase. Recent studies have reported that COX‐2 and the downstream prostaglandin E_2_ (PGE_2_) play a role in regulating the abundance and function of immune cells, such as CD8^+^, CD4^+^ T cells, and Treg cells, leading to tumor cell evasion of host antitumor immunity.^[^ ^8^ ^]^ In addition, COX‐2 and downstream PGE_2_ also drive constitutive Indoleamine 2,3 dioxygenase 1 (IDO1) expression in human tumors and subsequently mediate intrinsic immune resistance.^[^ ^9^ ^]^ Therefore, the COX‐2 inhibitor is an ideal PDT synergistic therapeutic agent for tumor treatment via PGE_2_ and IDO1 inhibition. Meanwhile, a reasonable combination of photosensitizer and COX‐2 inhibitor for joint use is the key to achieving synergistic effects. A commonly used strategy in the field of biomaterials is the coloading of other adjuvant drugs or biomacromolecules (e.g., proteins and nucleic acids) with photosensitizers into inorganic nanoparticles to enhance the therapeutic effect (all‐in‐one strategy). Common photosensitizers (e.g., Methylene blue (MB) and [Ru(bpy)_3_]^2+^] are positively charged compounds, whereas commonly used COX‐2 inhibitors are carboxyl‐containing compounds. It is difficult to achieve the loading of two drugs with different charges using the same nanomaterial, which also demonstrates the invalid “all‐in‐one strategy.” Moreover, the direct administration of COX inhibitors may result in a high blood concentration, potentially leading to common side effects such as severe gastrointestinal toxicity and thrombosis.^[^ ^10^ ^]^ Interestingly, an activated prodrug, triggered on demand by disease‐related factors to release the COX inhibitors, could effectively mitigate the potentially toxic side effects associated with a separate administration.

**Scheme 1:**
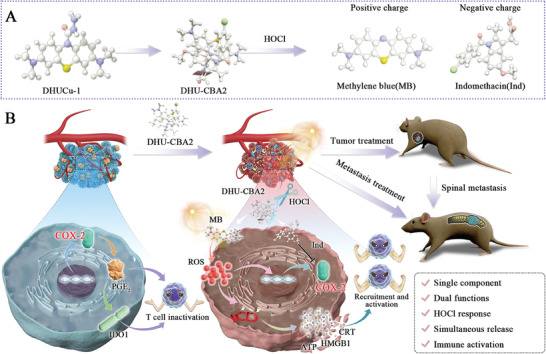
Schematic of the preparation and use of DHU‐CBA2 for treating lung cancer and spinal metastasis. A) Synthesis routes and HOCl‐response release of DHU‐CBA2. B) Conceptual and effective mechanisms of DHU‐CBA2 in triggering immunogenic cell death and blocking immune escape to remodel the tumor microenvironment.

To address these issues, in this study, we utilized a single‐component dual‐functional prodrug strategy to develop a system in which the photosensitizer MB and a COX‐2 inhibitor were activated by tumor‐specific microenvironment‐generated hypochlorous acid (HOCl). Using this prodrug, we investigated the immune activation mechanism of combined treatment (PDT and COX‐2 inhibitor) and found that the COX‐2 inhibitor could effectively inhibit the side effects of PDT. In summary, we successfully designed a single‐component, dual‐function autologous strategy for treating subcutaneous lung cancer and spinal metastasis. (**Scheme** **1**)

## 2 Results and Discussion

### 2.1 Design and Synthesis of DHU‐CBA2

We first screened for suitable drugs to develop a combination therapy for lung cancer using a photosensitizer and COX‐2 inhibitor. MB, a commonly used small‐molecule photosensitizer, has shown potential for antitumor applications owing to its high quantum yield for the generation of reactive oxygen species (ROS) and its biological safety. Extensive research has been conducted on the structure of MB to develop prodrugs that utilize its photosensitizing properties and function as a switch. Non‐steroidal anti‐inflammatory drugs (NSAIDs), including the commonly used COX‐2 inhibitor indomethacin (Ind), have anti‐inflammatory properties and are widely used in multiple fields. We hypothesized that a prodrug strategy could combine MB with Ind to develop an activated prodrug. We synthesized DHU‐CBA2, which contains acyl hydrazide as the responsive group. In order to emphasize the function of released indomethacin from DHU‐CBA2, we designed a compared prodrug (DHU‐CBA3). In fact, both DHU‐CBA2 and DHU‐CBA3 could release MB after the activation of HOCl. After a specific HOCl response, DHU‐CBA3 released the p‐toluic acid (pTC) but not indomethacin and thus showed no function of COX‐2 inhibition.

### 2.2 HOCl‐Response Behavior In Vitro

**Figure 1:**
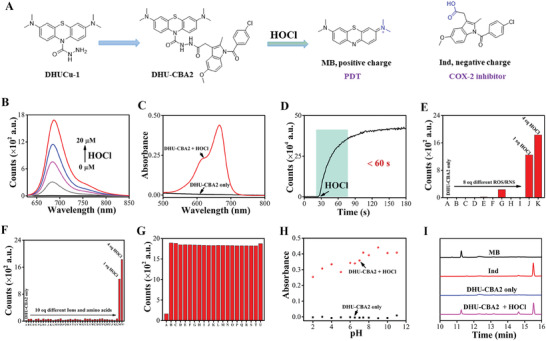
A) Schematic of the single‐component dual‐functional autoboost strategy. B) Fluorescence spectra of DHU‐CBA2 (5 µM) with different concentrations of HOCl. C) Absorption spectra of DHU‐CBA2 (5 µM in 10 mM PBS, pH 7.4) before and after the addition of 20 µM HOCl. D) Time‐dependent changes in fluorescent intensity of DHU‐CBA2 (5 µM) at 686 nm after adding HOCl (20 µM). E) Fluorescent intensity of DHU‐CBA2 (5 µM) at 686 nm after adding different ROS/RNS (40 µM) (from A to K: DHU‐CBA2 only, H_2_O_2_, TBHP, ROO•, NO, •OH, ONOO^–^, TBO•, O^2–^, 5 µM HOCl, and 20 µM HOCl). F) Fluorescent intensity of DHU‐CBA2 (5 µM) at 686 nm after adding different ions/amino acids (50 µM) (from A to N’: DHU‐CBA2 only, CH_3_COO^–^, CO_3_^2–^, SO_4_^2–^, F^–^, Cl^–^, I^–^, NO_2_^–^, S_2_O_3_^2–^, NH_4_^+^, Na^+^, Mg^2+^, Al^3+^, K^+^, Ca^2+^, Fe^3+^, Cu^2+^, Ni^2+^, Leu, Pro, Gly, Gln, Glu, Met, Lys, Trp, Ser, Thr, Asp, Ile, Val, His, Ala, Cys, Phe, Asn, Tyr, Arg, 5 µM HOCl, and 20 µM HOCl). G) Fluorescence intensity of DHU‐CBA2 (5 µM) upon addition of four equiv. HOCl in the presence of 4 equiv. various amino acids (from A to U: DHU‐CBA2 only, Leu, Pro, Gly, Gln, Glu, Met, Lys, Trp, Ser, Thr, Asp, Ile, Val, His, Ala, Cys, Phe, Asn, Tyr, Arg). H) The absorbance of DHU‐CBA2 (5 µM) at 664 nm before/after addition of 20 µM HOCl in buffer with different pH. I) HPLC analysis of 5 µM MB, 5 µM free Ind, 5 µM DHU‐CBA2 only, and 5 µM DHU‐CBA2 + 20 µM HOCl in 10 mM PBS (at 254 nm).

After synthesizing the compounds, we immediately investigated their response behaviors under physiological conditions (PBS buffer 10 mM, pH 7.4, containing 0.1% DMF as a co‐solvent). As shown in **Figure** **1**A, DHU‐CBA2 responded to HOCl, which was highly expressed in the tumor area. With the production of MB, the fluorescence intensity at 686 nm and absorbance of the prodrug at 664 nm were significantly enhanced (Figure 1B,C). This compound displayed good sensitivity and reached equilibrium within 60 s (Figure 1D). Moreover, DHU‐CBA2 displayed good selectivity toward other types of ROS/RNS (Figure 1E), cations, anions, and amino acids (Figure 1F), and neither of these analytes caused significant fluorescence changes in DHU‐CBA2. DHU‐CBA2 not only does not react with these substances but could also react with HOCl normally in the presence of these substances (Figure 1G). In the different pH (pH 2–12), DHU‐CBA2 also responded to HOCl (Figure 1H). DHU‐CBA3 exhibited a performance similar to that of DHU‐CBA2 and rapidly reacted with HOCl. These data indicate that this compound can be applied to tumor areas. Then, we investigated whether DHU‐CBA2 could release the corresponding drugs after the response. Therefore, we analyzed the reaction substrates using HPLC and found that DHU‐CBA2 displayed high drug release efficiency (Figure 1I). These data suggest that DHU‐CBA2 has good in vitro application performance, could be used to explore the interaction between photosensitizers and COX‐2 inhibitors in lung cancer, and could be used for tumor treatment.

### 2.3 HOCl‐Response Behavior in Biosystem

**Figure 2:**
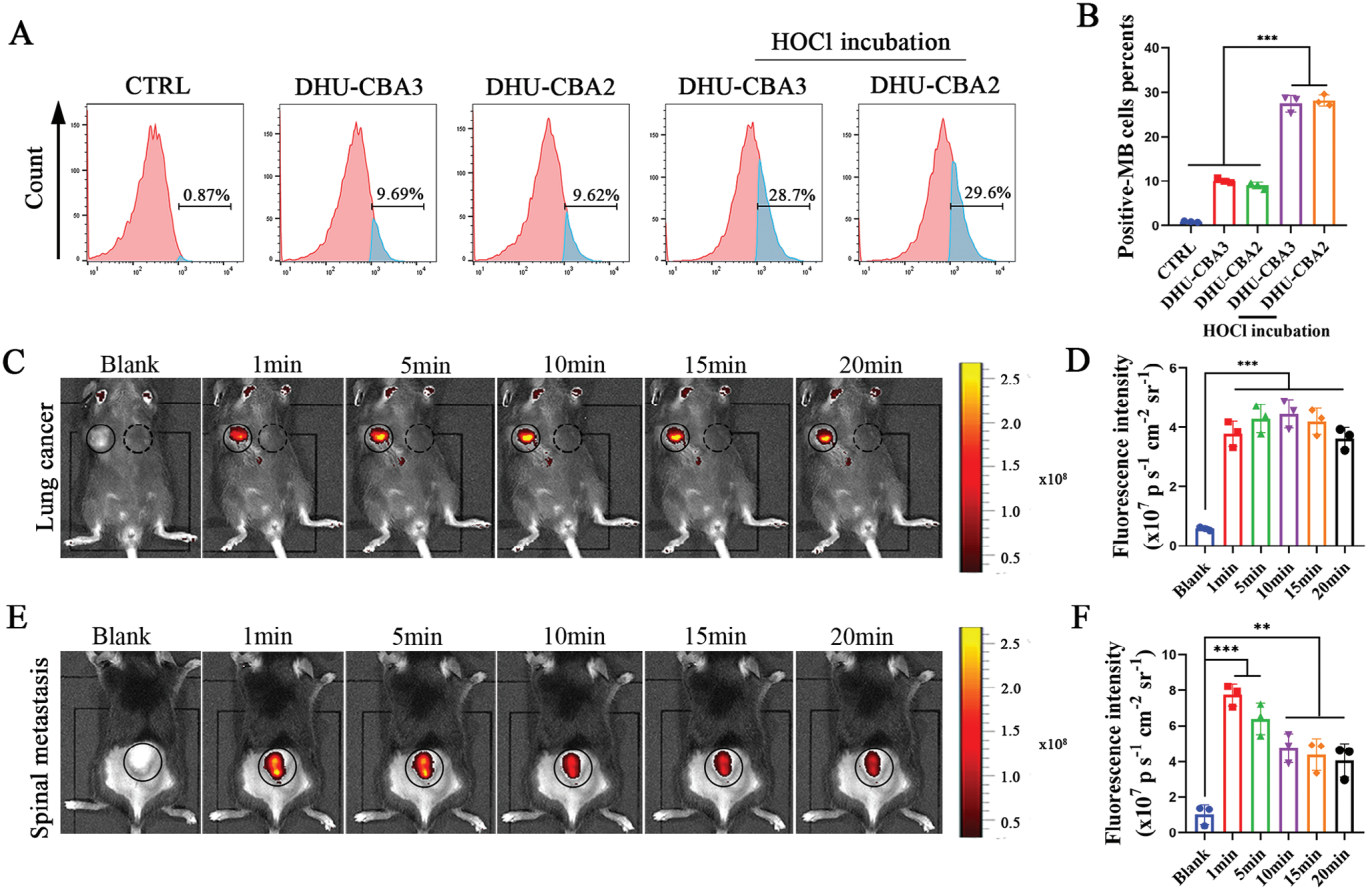
HOCl‐response behavior of DHU‐CBA2 in vitro and in vivo. After incubation with DHU‐CBA2, the medium was replaced by PBS with HOCl for 15 min. A) The MB‐positive LLC cells were detected by flow cytometry after various treatments, and (B) were further quantitatively analyzed. Experimental data in B) were presented as mean ± SD. Statistical significance was calculated via one‐way ANOVA with Tukey's test. (*n* = 3 replicates; ^*^ *p* < 0.05, ^**^ *p* < 0.01, ^***^ *p* < 0.001). C) The HOCl‐response behavior and (D) statistical analysis of DHU‐CBA2 were recorded by in vivo bioluminescence imaging in LLC‐bearing subcutaneous tumors. Experimental data in D) were presented as mean ± SD. Statistical significance was performed via one‐way ANOVA with Tukey's test. (*n* = 3 individual animals; ^*^ *p* < 0.05, ^**^ *p* < 0.01, ^***^ *p* < 0.001). E) The HOCl‐response behavior and F) statistical analysis of DHU‐CBA2 were recorded by in vivo bioluminescence imaging in LLC‐bearing subcutaneous tumors in spinal metastasis. Experimental data in (F) were presented as mean ± SD. Statistical significance was performed via one‐way ANOVA with Tukey's test. (*n* = 3 individual animals; ^*^ *p* < 0.05, ^**^ *p* < 0.01, ^***^ *p* < 0.001).

As a favorable drug release platform, the efficiency and speed of response should be evaluated in vivo and in vitro. High concentrations of ROS,^[^ ^11^ ^]^ especially HOCl,^[^ ^12^ ^]^ are a critical feature of solid tumors and are used as a typical trigger to activate drug platforms for cancer therapy.^[^ ^13^ ^]^ Given that MB has reliable fluorescence properties, the fluorescence intensity of the released MB was considered to guide the HOCl‐response behavior in vivo and in vitro.^[^ ^14^ ^]^ First, cancerous‐cell fluorescence imaging experiments in vitro were conducted to analyze the cellular uptake and intracellular activation behavior of this platform (10 µM) using flow cytometry (FCM) and confocal laser scanning microscopy (CLSM). After incubation with DHU‐CBA2 and DHU‐CBA3, MB‐positive LLC cells were significantly elevated in the groups with HOCl compared to the groups without HOCl (**Figure** **2**A,B; Figure S1, Supporting Information), indicating that the prodrugs could be efficiently ingested and further activated via external HOCl in vitro. Meanwhile, MB fluorescence in the DHU‐CBA2 group was not significantly different from that in the DHU‐CBA3 group in LLC cells, indicating a similar HOCl response behavior and efficiency of DHU‐CBA3 and DHU‐CBA2. Hence, in subsequent experiments, DHU‐CBA3 was considered an optimal control prodrug without NSAIDs release.

Second, fluorescence imaging of the tumor region in vivo was conducted to analyze the HOCl‐response of DHU‐CBA2 in the TME. MB fluorescence rapidly appeared in the right subcutaneous tumor area but not in the left subcutaneous non‐tumor area after injection (Figure 2C,D). The fluorescence intensity showed a slight decrease within 20 min. High concentrations of HOCl are widely known in normal solid tumors. However, it is not clear whether HOCl can reach a valid concentration to activate this platform in lung cancer spinal metastasis (LC‐SM). As shown in Figure 2E,F, the fluorescence peak was rapidly observed 1 min after intra‐LC‐SM injection, which was brighter than that in the subcutaneous tumor area, indicating the rapid and efficient response capacity of DHU‐CBA2 in the LC‐SM. The fluorescence intensity after intratumoral injection declined after 5 min, but the fluorescence intensity between 10 and 20 min was similar to that in the subcutaneous tumor area, indicating a more efficient HOCl response behavior of DHU‐CBA2 in the LC‐SM. These results revealed the good HOCl‐response and drug release behavior of DHU‐CBA2 in LLC tumor cells in vitro and in the TME of LC and LC‐SM in vivo.

### 2.4 Antitumor and ICD Induction Capacity of DHU‐CBA2 via PDT In Vitro

**Figure 3:**
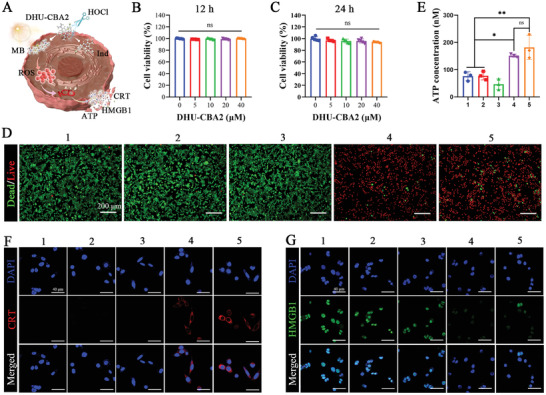
The cell killing and ICD induction capacity of activated DHU‐CBA2 with PDT in vitro. A) Schematic of the antitumor and ICD induction of DHU‐CBA2 with PDT in cancerous cells. The CCK8 results of the cell viability in LLC cells incubated with DHU‐CBA2 at different concentrations for 12 h B) and 24 h C). Experimental data in (B,C) were presented as mean ± SD. Statistical significance was calculated via one‐way ANOVA with Tukey's test. (*n* = 5 replicates, ns represents no significant difference). D) Live/dead cell staining assay of LLC cells after treatment by various treatments (1: CTRL; 2: DHU‐CBA3+HOCl; 3: DHU‐CBA2+HOCl; 4: DHU‐CBA3+HOCl (+); 5: DHU‐CBA2+HOCl (+); “(+)” represents high‐dose laser irradiation). E) Quantitative determination of ATP secretion in the cell culture supernatant after various treatments. Experimental data were presented as mean ± SD. Statistical significance was calculated via one‐way ANOVA with Tukey's test. (*n* = 3 replicates; ^*^ *p* < 0.05, ^**^ *p* < 0.01, ^***^ *p* < 0.001). F,G) Immunofluorescence staining results of CRT exposure and HMGB1 release in LLC cells after various treatments.

Subsequently, DHU‐CBA2 was used in the antitumor and ICD induction experiments with PDT in vitro; also, the conceptual mechanism is shown in **Figure** **3**A. The cytotoxicity assay showed LLC cells exhibited negligible cytotoxicity after being incubated with DHU‐CBA2 at different concentrations for 12 and 24 h (Figure 3B,C). Then, DHU‐CBA2 and DHU‐CBA3 (30 µM) were preincubated with HOCl (100 µM) to mimic prodrug activation in the tumor microenvironment. In order to show the favorable capacity of tumor killing and immunogenicity induction, we used high‐dose PDT but not low‐dose PDT in vitro in this part. (+) represents high‐dose PDT. As expected, negligible cytotoxicity toward LLC cells was observed in the dark environment via live/dead cell staining (Figure 3D). In contrast, the dead cells (red) occurred, and adherent living cells (green) were absent in the PDT groups, exhibiting the favorable antitumor capacity of HOCl‐activated DHU‐CBA2 and HOCl‐activated DHU‐CBA3 with PDT. Nevertheless, there was similar phototoxicity in the DHU‐CBA2+HOCl (+) group and the DHU‐CBA3+HOCl (+) group, indicating that the released indomethacin did not significantly enhance the antitumor capacity of PDT in vitro. The above results are consistent with those of a previous study.^[^ ^6e^ ^]^ To further investigate the capacity of ICD induction, the three main markers, namely, adenosine triphosphate (ATP) calreticulin (CRT), and high mobility group protein B1 (HMGB1), as the key “eat‐me signals” molecules, were next assessed in detail. As expected, cells treated with HOCl‐activated DHU‐CBA2 (or HOCl‐activated DHU‐CBA3) and laser irradiation secreted more ATP into the supernatant than untreated cells (Figure 3E). Immunofluorescence staining revealed that more CRT was exposed to the cellular surface, and more HMGB1 was secreted from the nucleus in the DHU‐CBA2+HOCl (+) and DHU‐CBA3+HOCl (+) groups (Figure 3F,G). In addition, there was no significant difference between the DHU‐CBA2+HOCl (+) and DHU‐CBA3+HOCl (+) groups, indicating that the released indomethacin did not enhance the capacity of PDT‐induced ICD. These results revealed that HOCl‐activated DHU‐CBA2 with PDT exhibited an excellent capacity for ICD induction. However, the released indomethacin did not play a role in this process.

### 2.5 Activated DHU‐CBA2 Eliminates PDT‐Induced PGE2 Expression

**Figure 4:**
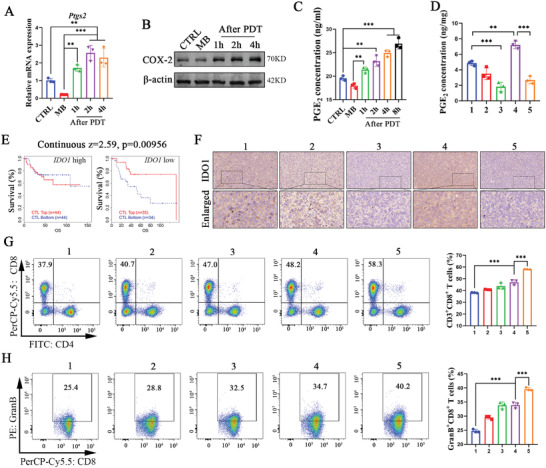
Immune response mechanism of DHU‐CBA2 with PDT via alleviating the high concentration of PGE_2_ and IDO1. A) Relative mRNA levels of *Ptgs2*, B) protein level of COX‐2, and C) the level of PGE_2_ in the supernatant in LLC cells with MB (+). (+) represents low‐dose laser irradiation in vitro. Experimental data in (A and C) were presented as mean ± SD. Statistical significance was calculated via one‐way ANOVA with Tukey's test. (*n* = 3 replicates, ^*^ *p* < 0.05, ^**^ *p* < 0.01, ^***^ *p* < 0.001). D) The protein level of PGE_2_ in LLC‐bearing subcutaneous tumors by various treatments (1: CTRL; 2: DHU‐CBA3; 3: DHU‐CBA2; 4: DHU‐CBA3 (+); 5: DHU‐CBA2 (+); “(+)” represents laser irradiation). Experimental data were presented as mean ± SD. Statistical significance was calculated via one‐way ANOVA with Tukey's test. (*n* = 3 individual animals per group, ^*^ *p* < 0.05, ^**^ *p* < 0.01, ^***^ *p* < 0.001). E) Association between CTL levels and overall survival of patients with lung cancer with different *IDO1* gene copy numbers. Continuous z = 2.59; P = 0.00956. Z‐scores and *p*‐values were computed by the two‐sided Wald test in Cox‐PH regression. F) Representative images of IDO1 expression in subcutaneous tumors after various treatments by immunohistochemistry staining analysis. G) CD3^+^CD8^+^, and H) GranB^+^CD8^+^ T cells in subcutaneous tumors after various treatments were detected and quantified by flow cytometry. Experimental data in (G,H) were presented as mean ± SD. Statistical significance was calculated via one‐way ANOVA with Tukey's test. (*n* = 3 individual animals per group, ^*^ *p* < 0.05, ^**^ *p* < 0.01, ^***^ *p* < 0.001).

PDT, as an emerging non‐invasive treatment, has attracted extensive attention in cancer therapy.^[^ ^15^ ^]^ In light of the ICD induction capacity, DHU‐CBA2 with a laser might initiate antitumor immune responses via PDT.^[^ ^6^, ^16^ ^]^ However, some byproducts of PDT may severely impair the immune response and the antitumor efficacy of PDT. Recent studies have uncovered a pivotal role for the expression and activity of cancerous COX‐2 expression and the downstream prostaglandin E_2_ (PGE_2_) in reshaping the inflammatory TEM and stimulating tumor progression through immune escape.^[^ ^8^, ^9^, ^17^ ^]^ *Ptgs2* encodes prostaglandin synthetase cyclocase‐2 (COX‐2), which is a rate‐limiting enzyme in prostaglandin synthesis and therefore plays a unique role in regulating PGE_2_ synthesis. The stressed and alive cancer cells with the low‐dose PDT would increase the expression of COX‐2 and the downstream PGE_2_;^[^ ^6e^ ^]^ For better‐conducting mechanism research, low‐dose PDT was used in this experiment. As shown in **Figure** **4**A, the mRNA expression of *Ptgs2* was inhibited by MB in LLC cells but sharply upregulated after low‐dose PDT in vitro. The upregulation of COX‐2 protein was noticeable at different time points after PDT (Figure 4B; Figure S2, Supporting Information). We further verified the high concentrations of downstream PGE_2_ in the supernatant of LLC cells at different time points after PDT (Figure 4C). Furthermore, we demonstrated that the activated prodrug (DHU‐CBA3) promoted PGE_2_ synthesis via low‐dose PDT in vivo (Figure 4D). Hence, we confirmed that only PDT induces PGE_2_ expression by upregulating COX‐2 in vivo and in vitro, which may be a critical byproduct of PDT. More importantly, we observed that up‐expressed PGE_2_ in the DHU‐CBA3 (+) group was significantly reversed in the DHU‐CBA2 (+) group (Figure 4D) in vivo, indicating that the indomethacin released from activated DHU‐CBA2 in vivo effectively eliminated PDT‐induced PGE_2_ expression.

### 2.6 Activated DHU‐CBA2 Eliminates High Expression of IDO1 In Vivo

IDO1 is a pivotal enzyme that catalyzes tryptophan degradation and kynurenine accumulation and is one of the most important metabolic pathways.^[^ ^6^, ^18^ ^]^ It is well‐known that increased expression has been identified in various solid tumors. Constitutive expression of IDO1 is driven by COX‐2 and its product (PGE_2_) via the MAPK, PKC, and PI3K cell signaling pathways.^[^ ^9^ ^]^ COX‐2 and PGE_2_‐induced high levels of IDO1 contribute to the establishment of immunosuppressive TEM. To better confirm the mechanism of IDO1‐associated immune evasion, we first validated the clinical relevance by predicting clinical outcomes from genomic (PRECOG) profiles containing nearly 30000 expression profiles from 166 cancers covering distinct histology. Survival data for lung cancer were acquired from Roepman_LungCancer@PRECOG. High‐infiltrated cytotoxic T lymphocytes (CTL) in tumor sites is widely associated with prolonged overall survival.^[^ ^19^ ^]^ Interestingly, we observed that this phenomenon was absent in lung cancer patients with higher *IDO1* expression but was present in lung cancer patients with lower *IDO1* expression (Figure 4E), supporting the theory that *IDO1* expression is associated with CTL dysfunction. Fortunately, after treatments, significantly decreased IDO1 expression was observed in the DHU‐CBA2 and DHU‐CBA2 (+) groups compared to that in the CTRL, DHU‐CBA3, and DHU‐CBA3 (+) groups (Figure 4F), indicating the favorable IDO1 downregulation capacity of DHU‐CBA2 via the release of indomethacin in vivo.

### 2.7 The Formidable Immune Activation Capacity of DHU‐CBA2 with PDT In Vivo

Previous studies have reported that COX‐2 inhibitors have cancer targetability and direct anti‐inflammatory effects to enhance the antitumor capacity of PDT.^[^ ^20^ ^]^ However, few studies focused on the synergistic effect of PDT and COX‐2 inhibitors on immune activation. Given the capacity for ICD induction and PGE_2_ and IDO1 downregulation, we further determined T cell infiltration and activation after DHU‐CBA2 treatment with PDT. The time point of T cell detection should be highlighted here because if detection is performed too early, T cell infiltration is inadequate; otherwise, T cells are exhausted. The maturation and activation of T cells lag 3–7 d after PDT.^[^ ^21^ ^]^ Thus, we chose the sixth day to detect T cells by FCM after various treatments on days 1, 3, and 5. The gating strategy is shown in Figure S3 (Supporting Information). As shown in Figure 4G, a small number of CD8^+^ T cells were observed in LLC‐bearing tumors in CTRL and DHU‐CBA3 groups; however, more CD8^+^ T cells were observed in tumors in DHU‐CBA3 (+) groups. Meanwhile, among five groups, the largest number of CD8^+^ T cells appeared in the DHU‐CBA2 (+) group, showing that the indomethacin released from activated DHU‐CBA2 promoted CD8^+^ T cell infiltration. The above result of CD8^+^ T cell infiltration was similar to the previous study.^[^ ^22^ ^]^ In this study, we further observed that tumors from the DHU‐CBA2 (+) group showed favorable enrichment in a CTL cluster expressing Granzyme B, which was more significant than that in the DHU‐CBA3 (+) group (Figure 4H). This indicates that the indomethacin released from activated DHU‐CBA2 restrains the intratumoral accumulation of dysfunctional CD8^+^ T cells and further enhances the immune activation capacity of PDT. These results collectively revealed that activated DHU‐CBA2 (+) in vivo triggers robust antitumor immune capacity via the concomitant release of indomethacin and MB‐based PDT.

### 2.8 Antitumor Capacity of DHU‐CBA2 with PDT In Vivo

**Figure 5:**
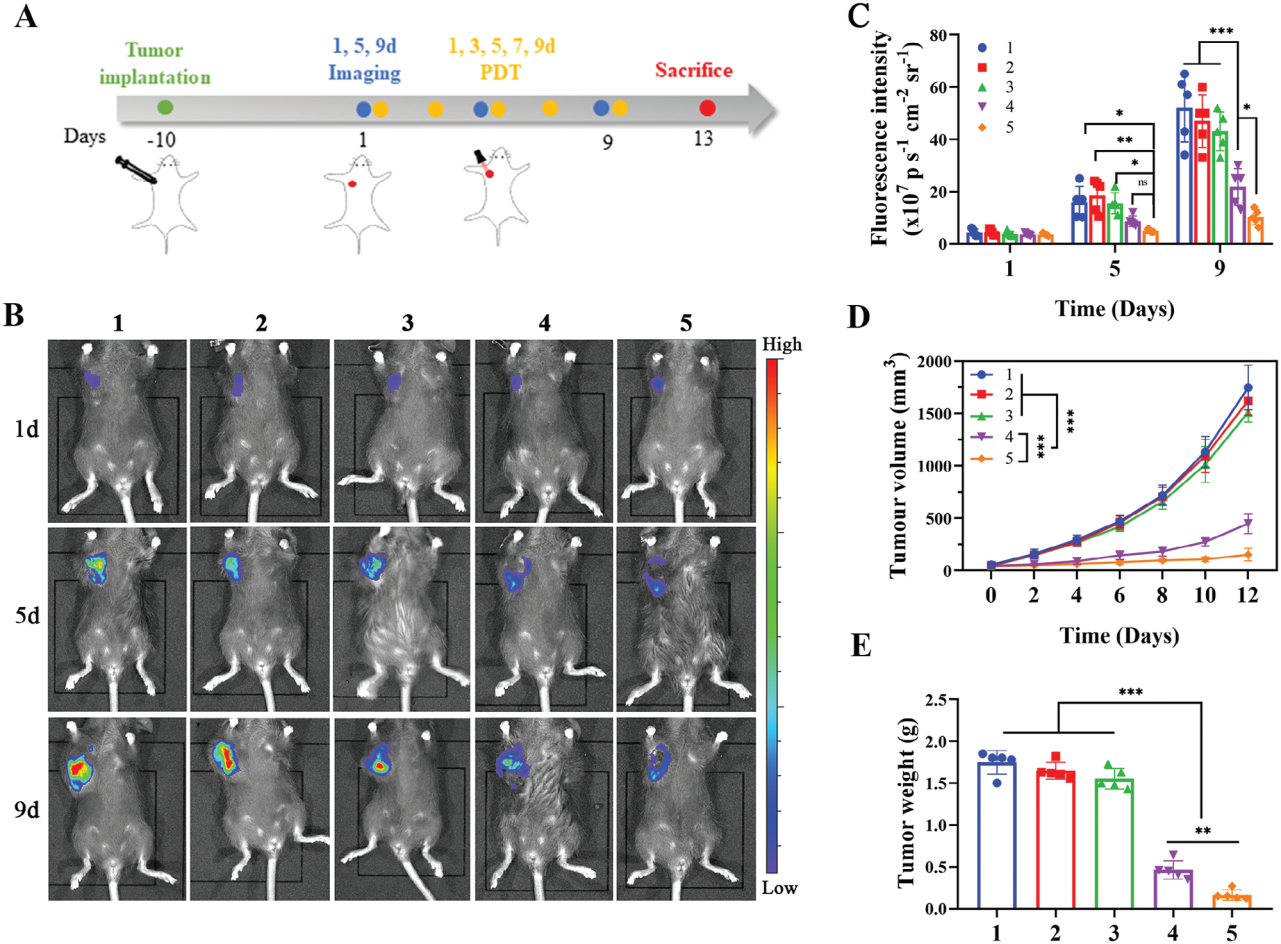
Therapeutic efficacy of DHU‐CBA2 in subcutaneous LLC‐bearing mice. A–E) Subcutaneous tumors by various treatments (1: CTRL; 2: DHU‐CBA3; 3: DHU‐CBA2; 4: DHU‐CBA3 (+); 5: DHU‐CBA2 (+); “(+)” represents laser irradiation): A) Schematic, B) bioluminescence images, and C) statistical analysis of tumorigenesis. Experimental data in C) were presented as mean ± SD. Statistical significance was calculated via two‐way ANOVA with Tukey's test. (*n* = 5 individual animals per group, ^*^ *p* < 0.05, ^**^ *p* < 0.01, ^***^ *p* < 0.001). (D) Tumor volume is recorded every two days. Experimental data were presented as mean ± SD. Statistical significance was calculated via two‐way ANOVA with Tukey's test. (*n* = 5 individual animals per group, ^*^ *p* < 0.05, ^**^ *p* < 0.01, ^***^ *p* < 0.001). (E) Tumor weight at the end of the observation period. Experimental data were presented as mean ± SD. Statistical significance was calculated via one‐way ANOVA with Tukey's test. (*n* = 5 individual animals per group, ^*^ *p* < 0.05, ^**^ *p* < 0.01, ^***^ *p* < 0.001).

Activated DHU‐CBA2 in vivo strengthened PDT‐induced immune activation via PGE_2_ and IDO1 inhibition, potentially improving the antitumor capacity of PDT. Thus, an antitumor schedule was established using LLC‐bearing subcutaneous models (**Figure** **5**A). Tumor growth was monitored using in vivo bioluminescence and measurements of tumor volume (Figure 5B–D). As anticipated, the fluorescence intensity and volume of the tumor in mice treated with PBS increased rapidly. DHU‐CBA3 and DHU‐CBA2 demonstrated slight tumor inhibition via released MB and indomethacin, perhaps because of the low level of immune activation (Figure 4G,H).^[^ ^8^, ^14^, ^23^ ^]^ However, the COX inhibitors might be useful adjuvant drugs to special tumor treatment (e.g., PDT) so long as prostanoids constitute a non‐negligible mean of immune escape during treatment.^[^ ^8a^ ^]^ Compared to DHU‐CBA3 with PDT treatment, DHU‐CBA2 with PDT treatment proved to be much more effective. Similarly, at the end of the treatment, tumor samples from each group were harvested and weighed. DHU‐CBA2 and DHU‐CBA3 showed slight tumor inhibition compared to PBS (Figure 5E). As expected, DHU‐CBA2 with PDT treatment exhibited more efficient tumor inhibition than DHU‐CBA3 with PDT treatment, which benefited from the release of indomethacin in vivo. Furthermore, we also demonstrated that only the laser group showed no tumor inhibition capacity compared to the PBS group (Figure S4, Supporting Information). These results revealed that the robust antitumor activity of DHU‐CBA2 resulted from the synergistic effects of PDT and indomethacin.

### 2.9 Toxicity Evaluation of DHU‐CBA2 In Vivo

A steady body weight gain was observed in DHU‐CBA2 (+) groups during the therapeutic period (Figure S5, Supporting Information). Subsequently, H&E staining revealed no pathological changes in major organs, including the heart, liver, spleen, lung, and kidney, in the DHU‐CBA2 (+) group compared with healthy mice (Figure S6, Supporting Information). Furthermore, biochemical indices (ALT, AST, and BUN) showed no significant differences between the healthy and DHU‐CBA2 (+) groups (Figure S7, Supporting Information), indicating negligible hepatotoxicity and nephrotoxicity. Finally, a complete blood count test showed that all indices of the DHU‐CBA2 (+) group were similar to those of the healthy group (Figure S8, Supporting Information). Thus, these results verify that DHU‐CBA2 (+) has excellent biocompatibility and serves as a safe treatment.

### 2.10 Anti‐SM Capacity of DHU‐CBA2 with PDT In Vivo

**Figure 6:**
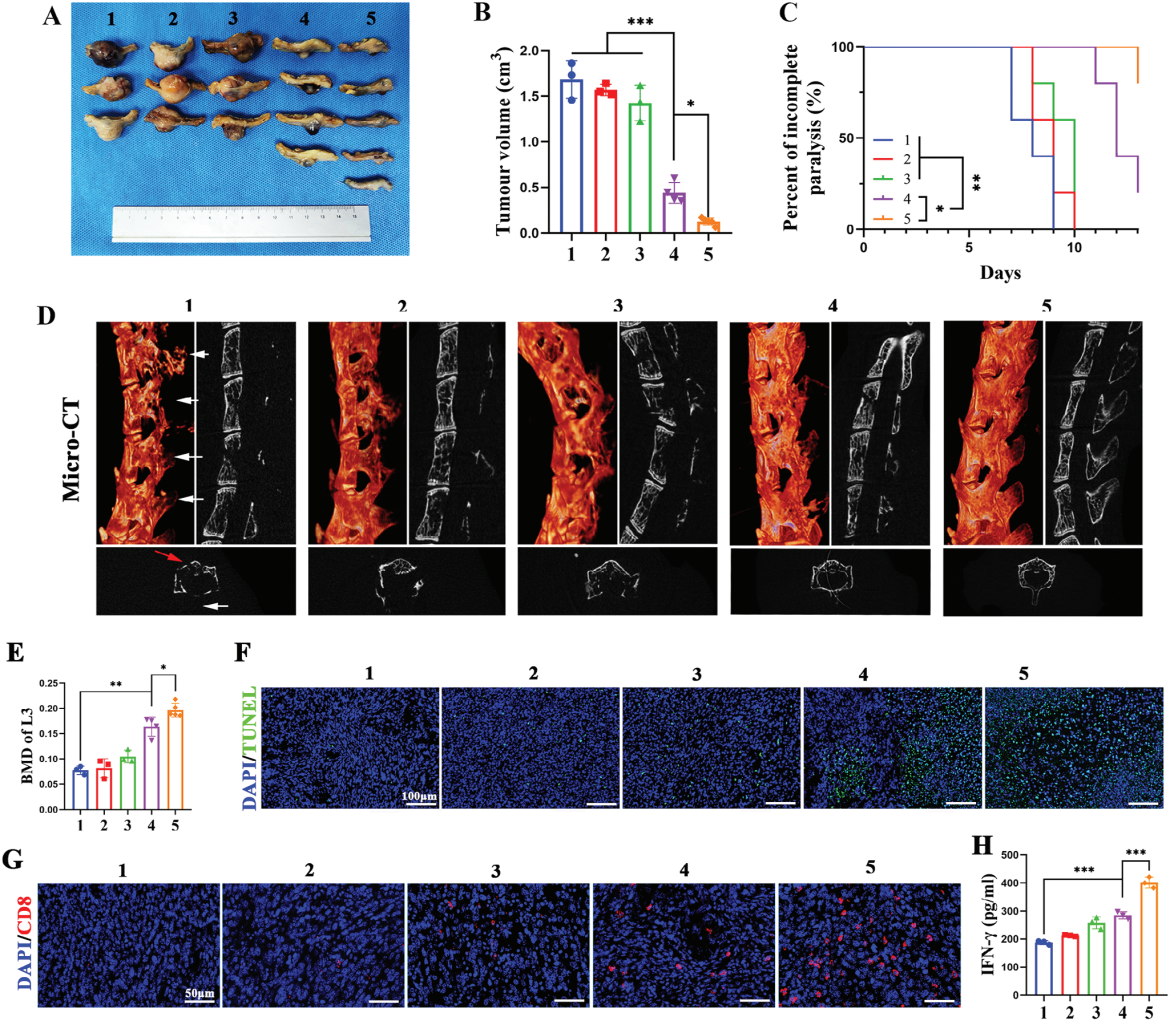
Therapeutic efficacy of DHU‐CBA2 in lung cancer spinal metastasis (LC‐SM). (A–H) LC‐SM tumors by various treatments (1: CTRL; 2: DHU‐CBA3; 3: DHU‐CBA2; 4: DHU‐CBA3 (+); 5: DHU‐CBA2 (+); “(+)” represents laser irradiation): A) Photographs of the tumors and B) tumor volume, at the end of the observation period. Experimental data in (B) were presented as mean ± SD. Statistical significance was calculated via one‐way ANOVA with Tukey's test. (*n* = 3–5 individual animals per group, ^*^ *p* < 0.05, ^**^ *p* < 0.01, ^***^ *p* < 0.001). C) Paralysis rate monitoring. Statistical significance was calculated via a log‐rank test for comparison. (*n* = 5 individual animals per group, ^*^ *p* < 0.05, ^**^ *p* < 0.01, ^***^ *p* < 0.001). D) 3D and planar view reconstruction images of spines showing the osteolytic vertebral plate (white arrow) and anterior centrum (red arrow) at the end of the observation period. (E) Bone mineral density (BMD) of lumbar three. Experimental data were presented as mean ± SD. Statistical significance was calculated via one‐way ANOVA with Tukey's test. (*n* = 3–5 individual animals per group, ^*^ *p* < 0.05, ^**^ *p* < 0.01, ^***^ *p* < 0.001). (F) TUNEL staining, (G) CD8^+^ T cells staining, and (H) IFN‐γ secretion. Experimental data in (H) were presented as mean ± SD. Statistical significance was calculated via one‐way ANOVA with Tukey's test. (*n* = 3 individual animals per group, ^*^ *p* < 0.05, ^**^ *p* < 0.01, ^***^ *p* < 0.001).

The spine is the most common site of lung cancer metastasis, causing pain, paralysis, and other symptoms that severely affect quality of life.^[^ ^24^ ^]^ Chemotherapy and surgery are common treatments that effectively alleviate symptoms but exhibit a limited capacity to extend life.^[^ ^24^, ^25^ ^]^ As a noninvasive treatment, PDT has few complications and brings hope of survival for lung cancer patients with SM. However, bone marrow is regarded as an immunosuppressive TEM^[^ ^26^ ^]^ resulting from elevated PGE_2_ production by bone mesenchymal stem cells (MSCs).^[^ ^27^ ^]^ Thus, a large amount of PGE_2_ from the dual generation of MSCs and PDT could induce a robust immunosuppressive TME. Our previous studies showed that SM showed more significant immunosuppressive TEM than primary lung cancer.^[^ ^28^ ^]^ Due to the superior immune activation ability, we first used the same therapeutic schedule as subcutaneous tumor treatment to evaluate the anti‐LC‐SM capacity of DHU‐CBA2 with PDT (Figure S9, Supporting Information). As shown in **Figure** **6**A,B, DHU‐CBA3 with PDT exhibited good tumor inhibition capacity, and DHU‐CBA2 with PDT exhibited the best tumor inhibition capacity. We also demonstrated that only the laser group showed no tumor inhibition capacity compared to PBS group (Figure S10, Supporting Information). In addition, DHU‐CBA2 (+) mice showed slight survival benefit (Figure S11, Supporting Information). According to weight monitoring, mice in the PBS, DHU‐CBA3, and DHU‐CBA2 groups showed significant weight loss after day 6 because of enlarged tumors and cachexia; however, mice in the DHU‐CBA3 (+) and DHU‐CBA2 (+) groups showed a steady weight gain because of their antitumor capacity (Figure S12, Supporting Information).

As spinal metastasis progresses, the growing tumor can compress the spinal cord, leading to paralysis, defecation issues, and other symptoms. Relieving tumor compression is often the preferred approach in clinical practice to alleviate these symptoms and improve the central nervous system. Therefore, to assess symptom improvement, we examined the incomplete paralysis rates in mice subjected to various treatments (Figure 6C). The incomplete paralysis of mice treated with PBS and DHU‐CBA3 started on day 7 and reached 100% by day 13. The incomplete paralysis rate in mice treated with DHU‐CBA2 showed a slight improvement. Significantly, the incomplete paralysis of mice in the DHU‐CBA2 (+) group was eliminated, which was more effective than that in the DHU‐CBA3 (+) group. Osteolysis is considered one of the leading causes of clinical symptoms such as pain, weakness, and paralysis. LC‐SM revealed severe bone destruction in the spine, including the spinous process, lamina, transverse process, and anterior centrum (Figure 6D; Figure S13, Supporting Information). The spine in DHU‐CBA3 (+) mice exhibited significant alleviation of bone destruction compared to that in the PBS group. The well‐preserved integrity of the spine with minor damage was observed in the DHU‐CBA2 (+) group. Quantitative analysis of bone mineral density (BMD) further confirmed this result (Figure 6E). These results revealed the lowest degree of spinal osteolysis after DHU‐CBA2 (+) treatment of LC‐SM.

Next, we investigated the antitumor immune response of LC‐SM. As expected, the TUNEL assay revealed that DHU‐CBA2 (+) cells displayed the best pro‐apoptotic function (Figure 6F). Immunofluorescence analysis showed that PDT effectively recruited CD8^+^ T cells via ICD induction in the DHU‐CBA3 (+) group, but the abundance of CD8^+^ T cells was the highest in the DHU‐CBA2 (+) group (Figure 6G). In addition, as shown in Figure 6H, the levels of antitumor cytokines (IFN‐γ) were the highest in the DHU‐CBA2 (+) group of five groups. Thus, DHU‐CBA2 (+) as an effective therapeutic method could activate a favorable immune response to strengthen the antitumor capacity of PDT, which was appreciable for LC‐SM treatment.

## 3 Conclusion

An HOCl‐responsive prodrug (DHU‐CBA2) was constructed through MB and indomethacin grafting. This prodrug has a good HOCl response to simultaneously release MB and indomethacin and maintains its pharmacological action in LC and LC‐SM. Thus, activated DHU‐CBA2 exhibited a favorable photodynamic performance in vitro and in vivo. PDT could efficiently induce ICD to recruit CD8^+^ T cells into the tumor region but inevitably drives COX‐2 and downstream PGE_2_ and IDO1 expression. Activated DHU‐CBA2 could inhibit the levels of PGE_2_ and IDO1 and further significantly strengthen the immune activation of PDT in vivo, including the recruitment and activation of CD8^+^ T cells; thus, DHU‐CBA2 apparently improved the antitumor capacity of PDT, both for LC and LC‐SM. The utilization of light and intratumoral injection in cancer therapy during in vivo experiments may present limitations, particularly for deep‐seated and visceral tumors. However, this study still introduces an innovative design strategy for releasing NSAIDs in response to HOCl. Additionally, it presents a novel treatment approach for enhancing the antitumor potential of PDT.

## Conflict of Interest

The authors declare no conflict of interest.

## Supporting information

Supporting information

## Data Availability

The data that support the findings of this study are available from the corresponding author upon reasonable request.
