# Supplementary material for: Single‐Component Dual‐Functional Autoboost Strategy by Dual Photodynamic and Cyclooxygenase‐2 Inhibition for Lung Cancer and Spinal Metastasis
Source: Adv Sci (Weinh). 2024 Jan 15;11(12):2303981. doi: 10.1002/advs.202303981 (PMC10966547; doi:10.1002/advs.202303981)
Supplement: Supplementary file 1 — Supporting information [file ADVS-11-2303981-s001.pdf]

ADVANCED SCIENCE
Open Access

Supporting Information

for *Adv. Sci.*, DOI 10.1002/advs.202303981

Single-Component Dual-Functional Autoboost Strategy by Dual Photodynamic and Cyclooxygenase-2 Inhibition for Lung Cancer and Spinal Metastasis

Ben Wang, Zhen-Ni Lu, Meng-Xiong Song, Xiao-Wen He, Zhi-Chao Hu, Hai-Feng Liang, Hong-Wei Lu, Qing Chen, Bing Liang, Tao Yi, Peng Wei*, Li-Bo Jiang* and Jian Dong**

Experimental section

Chemicals and materials: All the starting materials were obtained from commercial suppliers and used as received. General chemicals were purchased from Adamas-beta® and Sinopharm Chemical Reagent Co., Ltd.

Instrumentation and characterization: ^{1}H NMR (400 MHz) and ^{13}C NMR (100 MHz) spectra were obtained using a Bruker AV400 nuclear magnetic resonance spectrometer with DMSO-d_6 as the solvent. Proton chemical shifts are reported in parts per million downfield from tetramethylsilane (TMS), with tetramethylsilane (δ = 0.0 ppm) or DMSO-d_6 (39.52 ppm for ^{13}C) as the chemical shift standard. High-resolution mass spectra (HRMS) were obtained on a Bruker Micro TOF II instrument using electrospray ionization (ESI) and direct injection methods. UV-Vis absorption spectra were obtained using a Shimadzu UV-2600 spectrophotometer at a medium scanning rate and quartz cuvettes with a path length of 1 cm. Fluorescence spectra were recorded at room temperature using an Edinburgh FLS 1000 spectrometer with an Xe lamp as the excitation source. HPLC was performed using an Agilent 1200 Series HPLC system.

Synthesis of DHU-CBA2 and DHU-CBA3: DHUCu-1 was synthesized according to our reported procedure.[1] p-Toluic acid (0.22 g, 1.6 mmol, 1 eq) or Ind (0.56 g, 1.6 mmol, 1 eq) was dissolved in 10 mL CH_2Cl_2 and stirred at 0 °C, Oxalyl chloride (0.30 g, 2.4 mmol, 1.5 eq) was added dropwise, and the mixture was stirred in an ice-water bath for 0.5 h. The solvent was removed under reduced pressure. The obtained residue was dissolved in CH_2Cl_2 and added dropwise to a mixture of DHUCu-1 (0.5 g, 1.46 mmol, 1 equiv), 10 mL CH_2Cl_2, and Sodium Carbonate (0.46 g, 4.38 mmol, 3 equiv). The mixture was stirred in an ice-water bath for 0.5 h, and then stirred at room temperature until the reaction was complete (the progress of reaction was monitored by TLC). The solvent was removed under reduced pressure. The surplus was extracted with 3 × 100 mL of ethyl acetate (EtOAc), washed with brine, and dried over anhydrous sodium sulfate (Na_2SO_4). After removing Na_2SO_4 by filtration, the crude product was evaporated using a rotary evaporator. The resulting residue was purified using a silica gel column (EtOAc/petroleum ether = 1:1) to afford compound DHU-CBA2 and DHU-CBA3 as white solids in yields of 58% (0.39 g) and 66% (0.66 g), respectively. DHU-CBA2: ^{1}H NMR (400 MHz, DMSO-d_6) δ (ppm): 9.90 (s, 1H), 8.07 (s, 1H), 7.69–7.67 (m, 2H), 7.64–7.62 (m, 2H), 7.35 (d, J = 8.8 Hz, 2H), 7.19 (d, J = 2.4 Hz, 1H), 6.92 (d, J = 8.8 Hz, 1H), 6.71 (d, J = 2.4 Hz, 2H), 6.68–6.66 (m, 2H), 6.64 (d, J = 2.8 Hz, 1H), 3.77 (s, 3H), 3.53 (s, 2H), 2.88 (s, 12H), 2.25 (s, 3H) (Figure S14-S15). ^{13}C NMR (100 MHz, DMSO-d_6) δ (ppm): 169.4, 168.3, 156.0, 155.3, 149.1, 138.1, 135.7, 134.7, 133.3, 131.7, 131.3, 130.7, 129.5, 128.3, 127.4, 114.9, 114.3, 111.9, 111.7, 110.7, 102.6, 55.9, 40.7, 29.5, 13.9 (Figure S16-

S18). HRMS (ESI): $[M+Na]^+$ calculated for $C_{36}H_{35}ClN_6O_4SNa$:705.2021; found: 705.2003 (Figure S19). ^{1}H NMR (400 MHz, DMSO-d_6) δ (ppm): 10.20 (s, 1H), 8.46 (s, 1H), 7.77 (d, J = 7.6 Hz, 2H), 7.58 (d, J = 8.4 Hz, 2H), 7.29 (d, J = 7.6 Hz, 2H), 7.09 (s, 4H), 2.98 (s, 12H), 2.36 (s, 3H). (Figure S20-21). ^{13}C NMR (100 MHz, DMSO-d_6) δ (ppm): 166.4, 155.5, 149.1, 142.1, 133.4, 130.4, 129.4, 128.4, 127.9, 127.5, 111.8, 110.8, 40.7, 21.5 (Figure S22). HRMS (ESI): $[M+Na]^+$ calculated for $C_{25}H_{27}N_5O_2SNa$:484.1778; observed: 484.1775 (Figure S23).

Cells and animal model: The mouse lung cancer cell line (LLC cells) was purchased from the Chinese Academy of Sciences Cell Bank (Shanghai, China). LLC cells were cultured in a high-glucose DMEM medium containing 10% FBS and 1% streptomycin/penicillin in a 5% CO_2 incubator at 37 °C. Seven-week-old female C57BL/6 mice were purchased from JSJ (Shanghai, China). To establish the lung cancer (LC) models—1×10^6 LLC cells were subcutaneously injected into the right axillary region. To simulate lung cancer spinal metastases (LC-SM), we constructed a vertebral plate implantation model based on well-accepted methods for simulating LC-SMs.[2] Briefly, 1×10^6 LLC cells were injected into the lumbar vertebral plate of every mouse. All animal experiments were approved by the animal ethics committee of Zhongshan Hospital (No: 2023-279).

Cell viability assay: LLC cells were seeded overnight and incubated with complete medium containing DHU-CBA2 (0–40 μM) for 12 h and 24 h. After incubation, the CCK-8 reagent (10%) was co-incubated without light for nearly 1 h. Afterward, the plate was analyzed at an absorbance of 450 nm (A450). The cell viability was determined as follows: Cell viability (%) = $(A - A_0)/(As - A_0) \times 100\%$. A, As, and A_0 represent the A450 values in the experimental group with DHU-CBA2, the control group without DHU-CBA2, and the medium group, respectively.

Drug release in vitro and in vivo: The drug release properties of DHU-CBA2 and DHU-CBA3 *in vitro* and *in vivo* were studied in LLC cells and LLC-bearing models, respectively. To detect the drug release *in vitro,* LLC cells were incubated with or without 1 mL of DHU-CBA2 (10 μM) and DHU-CBA3 (10 μM) for 1 h. All groups were then reincubated with fresh PBS, and two groups with DHU-CBA2 and DHU-CBA3 were incubated with fresh PBS containing HOCl (50 μM) for 15 min. Finally, LLC cells were collected and analyzed using flow cytometry and confocal laser scanning microscopy (CLSM, Olympus, JPN). To detect the drug release behavior *in vivo,* DHU-CBA2 (1 mM) was intratumorally injected into the right axillary tumor area, and subcutaneously injected into left axillary non-tumor area in subcutaneous LLC-bearing models (n = 3 individual animals), followed by detection at various time points (1, 5, 10, 15, and 20 min) via *in vivo* bioluminescence imaging (IVIS Spectrum, PerkinElmer, USA). To detect the drug release behavior in lung cancer spinal metastasis *in vivo,* the

same intratumoral injection plan was used in the LC-SM models, and further was detected via *in vivo* bioluminescence imaging (n = 3 individual animals).

Live/Dead assay: A live/dead cell experiment was performed to show the anti-tumor capacity of DHU-CBA2 via high-dose PDT *in vitro*. The LLC cells seeded in the 6-well plate were incubated with DMEM medium, and then the medium in four groups was respectively added with DHU-CBA3 (30 µM) or DHU-CBA2 (30 µM) containing HOCl (100 µM). After incubation in a dark environment, the groups were washed with PBS and treated with or without 100 mW cm^{-2} laser irradiation for 10 min. Therefore, cells were divided into five groups according to different treatments, namely, CTRL, DHU-CBA3+HOCl, DHU-CBA2+HOCl, DHU-CBA3+HOCl (+), and DHU-CBA2+HOCl (+), where (+) represents laser treatment. Subsequently, LLC cells were stained with calcein AM and PI solutions for 30 min. Finally, live/dead cell imaging was performed using CLSM.

ICD marker detection after PDT in vitro: After cell adhesion to the confocal dish, the LLC cells were divided into five groups with various treatments. Four hours later, the cells were fixed for 15 min and permeabilized using 0.5% Triton X-100 for 5 min. After washing with PBS, the cells were blocked and then incubated overnight with primary antibodies against CRT and HMGB1. Subsequently, the cells were stained with second antibodies for 1 h in a dark environment at 37 ℃, and then were stained with DAPI. The stained cells were observed and imaged using CLSM. The ATP concentration in the supernatant was detected using an ATP Determination Kit (S0027, BYT, China) according to a standard protocol to quantify the extracellular ATP in the cell culture.

Quantification of Ptgs2 after PDT in vitro: To determine the effect of PDT on *Ptgs2* expression *in vitro*, LLC cells were cultured with 1 µM MB. After washing by new medium, LLC cells were exposed to the low-dose PDT with 660 nm laser (13 mW cm^{-2}) for 5min *in vitro*, then cultured for different periods. Afterward, the total RNA was extracted from the cells and tissues using a RNA-Quick Purification Kit (ES Science). RNA was then quantified and reverse-transcribed using a PrimeScript RT reagent kit. qPCR was performed using SYBR Premix Ex Taq in a QuantStudio PCR system. Additionally, the PCR primer sequences were provided as follows: *Ptgs2* (Fwd:5’-CTGGTGCCTGGTCTGATGATGTATG-3,’ Rev:5’-GGATGCTCCTGCTTGAGTATGTCG-3’); GAPDH (Fwd:5’-GGCAAATTCAACGGCACAGTCAAG-3,’ Rev:5’- TCGCTCCTGGAAGATGGTGATGG -3’).

Protein quantification of COX-2 and PGE_2 after PDT in vitro: After variouos treatment as described above, cells were lysed to acquire the protein samples with SDS-PAGE sample loading buffer, which then were boiled at 100 °C for 10 min. Equal protein samples were separated by 12% SDS-

polyacrylamide gels and then transferred to the 0.45 μm poly(1,1-difluoroethylene) (PVDF) membrane. Subsequently, the membranes were blocked and incubated overnight with primary antibodies for COX-2 and β-actin antibody. The next day, the membranes were incubated with a secondary antibody for 1 h. Finally, the membranes were exposed to the ECL reagent and quantitatively analyzed using the gray value. As for PGE_2 detection, PGE_2 released into the LLC cells supernatant were quantified using commercial ELISA kits with a standard protocol.

PGE_2 detection and IDO1 staining in vivo: Mice with subcutaneous tumors were intratumorally injected with PBS, DHU-CBA3 (1 mM), or DHU-CBA2 (1 mM) with or without low-dose PDT (50 mW cm^{-2}, 5 min) according to the schedule (see Figure 5A). At the end of the various treatments, tissues were harvested and detected by PGE_2 ELISA kit with a standard protocol. As for IDO1 staining by immunohistochemistry staining (IHC), tissues were harvested, fixed, and embedded. The samples were sliced and dewaxed, followed by antigen repair and sealing. Afterward, the slides were incubated with the primary anti-IDO1 antibody (13268-1-AP, proteintech, China) overnight at 4 ℃. Then, the secondary antibody was dripped onto the slides for 30 min at room temperature and finally detected using a DAB reaction.

Survival data analysis: To validate the clinical relevance, the association between IDO1 expression and survival information was publicly acquired from the Tumor Immune Dysfunction and Exclusion (TIDE) online database (http://tide.dfci.harvard.edu/query/)[3] using Roepman_LungCancer@PRECOG, covering adeno, large, and squamous subtypes.

Antitumor efficacy of DHU-CBA2 via PDT in vivo: When the tumor volume reached approximately 50-100 mm^3, the subcutaneous LLC-bearing mice were randomly divided into five groups (n = 5 individual animals per group) and intratumorally injected with related drugs (1 mM): PBS, DHU-CBA3, DHU-CBA2, DHU-CBA3 (+), and DHU-CBA2 (+). (+) represents high-dose PDT (660 nm laser, 400 mW cm^{-2}, 15 min). From the 0st day, the tumor volume were recorded every 2 days. Tumor volume was calculated as follows: width^2 × length/2. On the 1st, 5th, and 9th day, tumorigenesis in every group was monitored using *in vivo* bioluminescence imaging. On the 13th day, the mice were sacrificed, and tumor tissues were harvested. We then measured the tumor weigh. To investigate the biosafety of DHU-CBA2 (+) treatment, the main organs (heart, liver, spleen, lung, and kidney) of healthy mice group and DHU-CBA2 (+) group were collected for H&E staining, and body weight was recorded. Blood samples were collected for complete blood counts and biochemical analyses. Biochemical indices, including alanine transaminase (ALT), aspartate aminotransferase (AST), and blood urea nitrogen (BUN) were analyzed to determine the function of the liver and kidney.

The antitumor efficacy against LC-SM was evaluated using a LC-SM model (Figure S9, Supporting Information). From the 0th day, the LC-SM mice were randomly divided into five groups (n = 5 individual animals per group) and then intratumorally injected with related drugs (1 mM): PBS, DHU-CBA3, DHU-CBA2, DHU-CBA3 (+), and DHU-CBA2 (+). (+) represents high-dose PDT. The body weights of the mice were recorded every 2 days. The incomplete paralysis and survival rates in each group were recorded daily. Indications of humane end points requiring euthanasia: 1, severe emaciation (15-20%), 2, hypothermia, 3, complete paralysis of the lower limbs with stiffness, 4, bleeding or mucous secretions in any part of the body, 5, dyspnea, 6, tumor ulceration, 7, obvious abdominal enlargement, 8, loss of appetite, 9, exophthalmia, 10, seizures. At 13th day, the tumor tissues were harvested and photographed, and the tumor volume was calculated. The resected spines were analyzed using micro-CT scanner SkyScan 1176.

In addition, in order to demonstrate the effect of laser on tumor inhibition, the subcutaneous LLC-bearing mice and LC-SM mice respectively were intratumorally injected with PBS, and randomly divided into two groups (n = 5 individual animals per group): PBS and PBS (+) group. (+) represents high-dose PDT. The mice received the same treatment plan as described above. At 13th day, the tumor tissues were harvested and photographed, and the tumor volume was calculated.

Activating antitumor immunity in vivo: First, the subcutaneous LLC-bearing mice were intratumorally injected with related drugs (1 mM) and treated as following (n=3 individual animals per group): PBS, DHU-CBA3, DHU-CBA2, DHU-CBA3 (+), or DHU-CBA2 (+). (+) represents high-dose PDT. Tissues were collected at 6th day after various treatments on days 1, 3 and 5. After digestion and filtration, the cell suspension was resuspended and blocked for 10 min. The cells were then stained with BV510 anti-mouse CD45, PE-Cy7 anti-mouse CD3, FITC anti-mouse CD4, PerCP-Cy5.5 anti-mouse CD8, and PE anti-mouse GranB in the dark to analyze the infiltration of $CD8^+$ ($CD3^+CD8^+$ $CD4^-$) T cells and the activation of $CD8^+$ T cells ($GranB^+CD8^+$) by FCM.

Subsequently, LC-SM mice with similar tumor volume were randomly divided into five groups (n = 3 individual animals per group), and treated as described above. Tissues were collected on the sixth day after various treatments on days 1, 3 and 5. We analyzed the pro-apoptotic efficacy of DHU-CBA2 with PDT for SM-bearing tumors using the TUNEL assay. To further analyze T-cell infiltration, tissues were evaluated by $CD8^+$ T cells immunofluorescence staining. The levels of IFN-γ in the serum of peripheral blood were measured using related ELISA kits.

Statistical analysis: All data were expressed as a mean ± standard deviation (SD) from at least three independent experiments or biological replicates. Data were analyzed with the following tests (see Fig. legends for details): Two-sided Student's *t*-test was used to analyze the statistical significance between the two groups; Analysis of variance (ANOVA) with a Tukey's test was used for multiple group comparisons; Log-rank test was performed to analyze the difference between paralysis and survival curves; Z-scores and p-values of survival analysis in TIDE database are computed by the two-sided Wald test in Cox-PH regression.[3b] All statistical tests were performed two-sided. *$p < 0.05$ (*$p < 0.05$, **$p < 0.01$, ***$p < 0.001$) were considered statistically significant. Statistical analyses were performed using GraphPad Prism 9 software.

Reference

[1] Y. Chen, Z. Long, C. Wang, J. Zhu, S. Wang, Y. Liu, P. Wei, T. Yi, *Dyes Pigm.* **2022**, 204, 110472.

[2] a) L. Zhou, H. Liang, Y. Ge, W. Ding, Q. Chen, T. Zhang, L. Xiao, Y. Li, J. Dong, X. He, F. Xue, L. Jiang, *Adv. Healthcare Mater.* **2022**, 11, 2200938; b) H. Liang, L. Zhou, Z. Hu, Y. Ge, T. Zhang, Q. Chen, B. Wang, S. Lu, W. Ding, J. Dong, F. Xue, L. Jiang, *Small* **2022**, 18, 2107787.

[3] a) J. Fu, K. Li, W. Zhang, C. Wan, J. Zhang, P. Jiang, X. S. Liu, *Genome Medicine* **2020**, 12, 21; b) P. Jiang, S. Gu, D. Pan, J. Fu, A. Sahu, X. Hu, Z. Li, N. Traugh, X. Bu, B. Li, J. Liu, G. J. Freeman, M. A. Brown, K. W. Wucherpfennig, X. S. Liu, *Nat. Med.* **2018**, 24, 1550.

Supplementary Figures

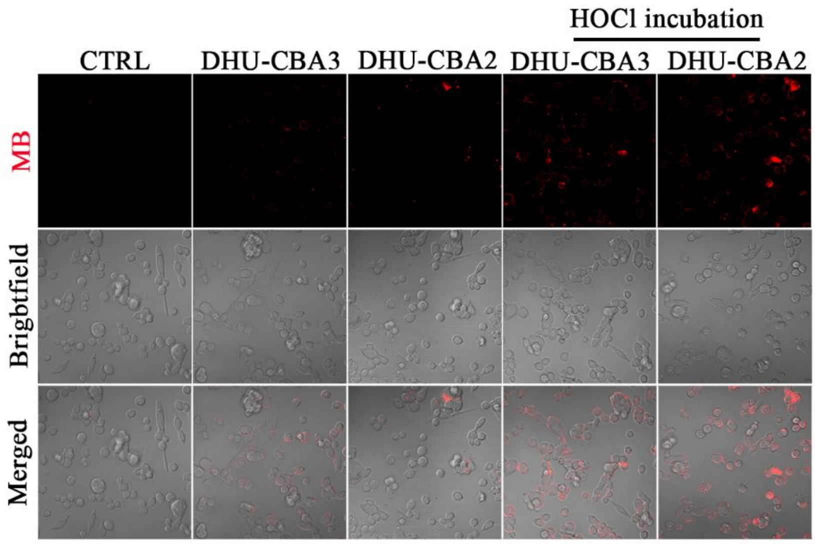

Figure S1. After incubation with the prodrugs for 1 h, the medium was replaced by PBS with HOCl for 15 min. Immunofluorescence staining results of DHU-CBA3 and DHU-CBA2 in the LLC cells with or without HOCl incubation.

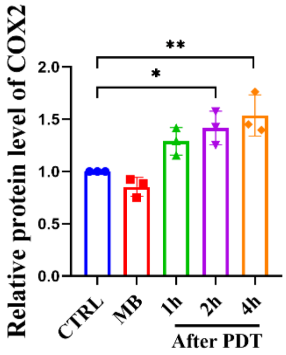

Figure S2. Quantitative analysis of WB results in Figure 4B. Experimental data were presented as mean ± SD. Statistical significance was calculated via one-way ANOVA with Tukey's test. (n=3 replicates, *$p < 0.05$, **$p < 0.01$, ***$p < 0.001$).

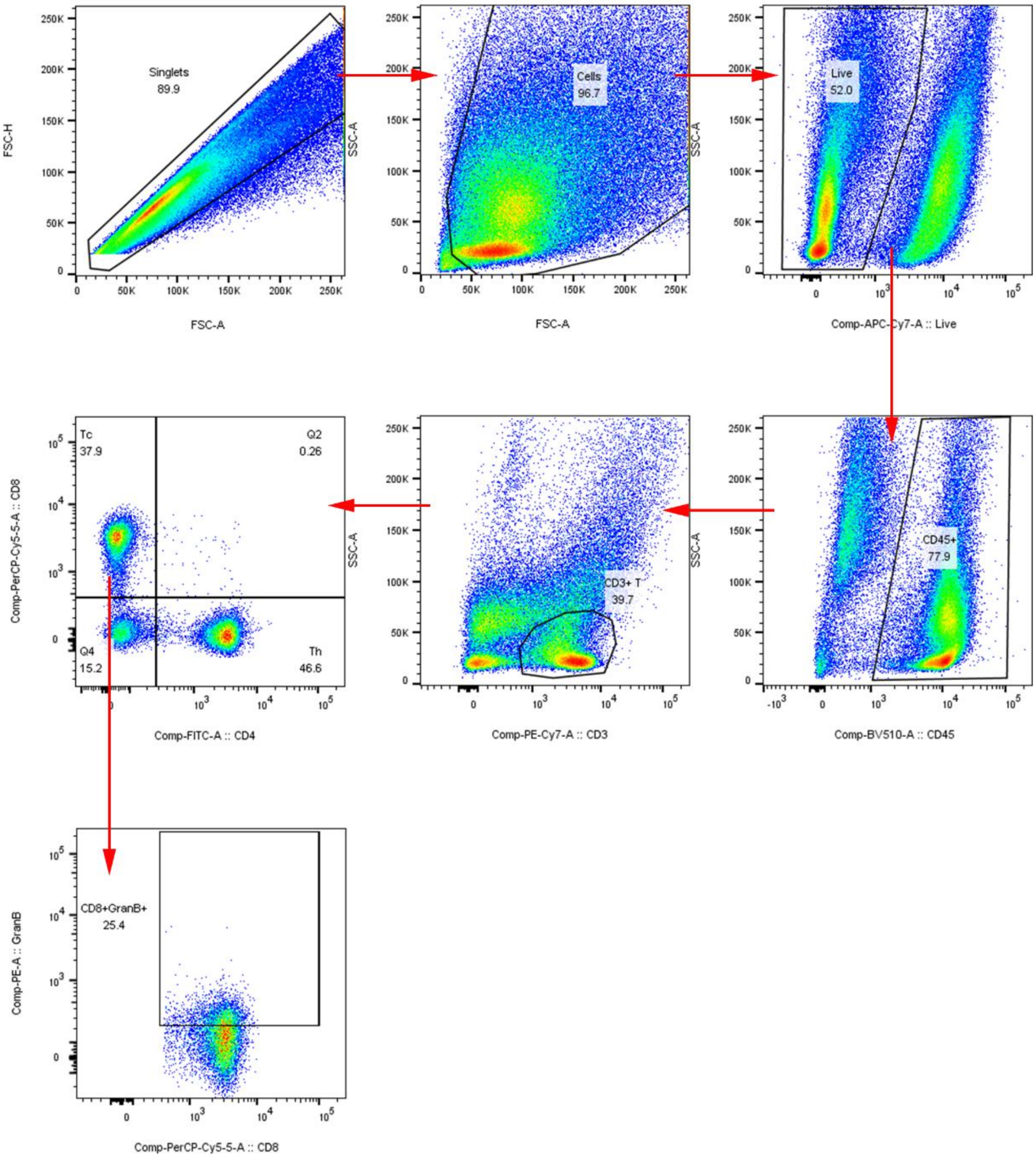

Figure S3. Flow cytometry gating strategy was conducted to ascertain immune cell populations in tumors. $CD8^+$ T cells were gated on $CD45^+CD3^+CD8^+CD4^-$ cells, and activated $CD8^+$ T cells were gated on $CD45^+CD3^+CD8^+GranB^+$ cells.

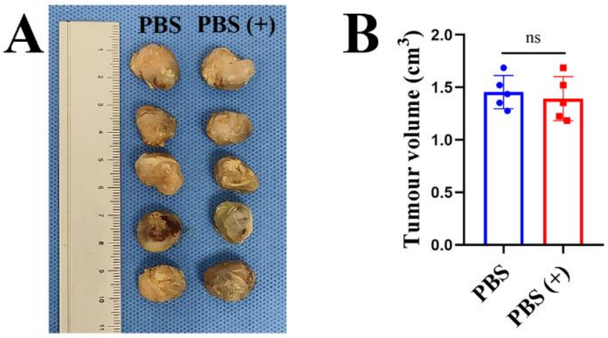

Figure S4. Photograph (A) and tumor volume (B) of subcutaneous tumors by various treatments at the end of the observation period. "(+)" represents laser irradiation). All data in (B) were presented as mean ± SD. Statistical significance was calculated via two-sided Student's *t*-test. (n=5 individual animals per group, ns represents no significant difference).

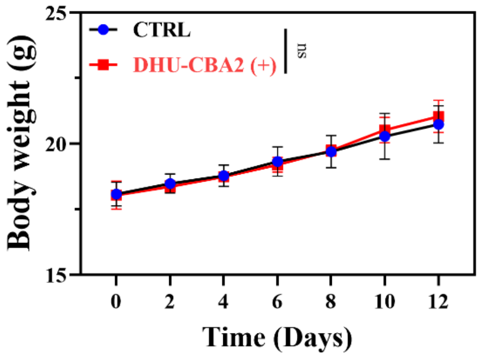

Figure S5. The body weight of healthy mice group and DHU-CBA2 (+) group. "(+)" represents laser irradiation). All data were presented as mean ± SD. Statistical significance was calculated two-way ANOVA with Tukey's test. (n=5 individual animals per group, ns represents no significant difference).

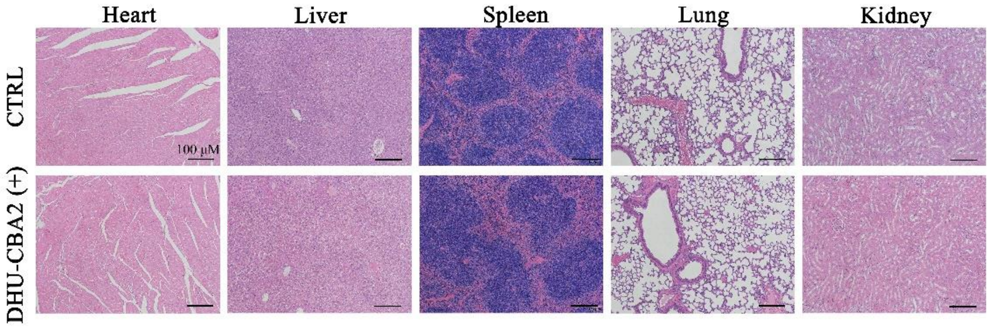

Figure S6. H&E staining of main organs of subcutaneous LLC-bearing mice after DHU-CBA2 (+) treatment. (+) represents laser irradiation.

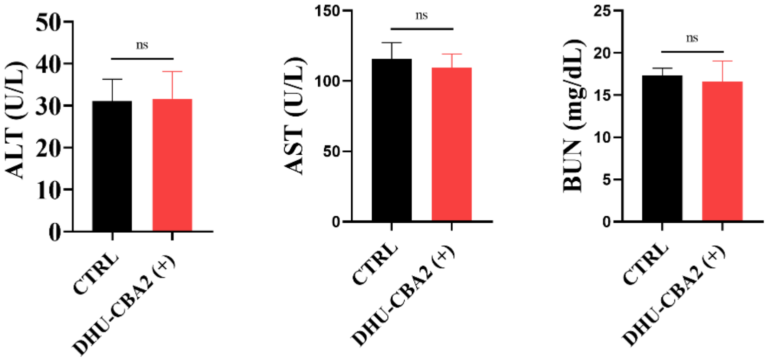

Figure S7. Plasma levels of ALT, AST, and BUN of subcutaneous LLC-bearing mice after DHU-CBA2 (+) treatment. (+) represents laser irradiation. All data were presented as mean ± SD. Statistical significance was calculated via two-sided Student's *t*-test. ns represents no significant difference.

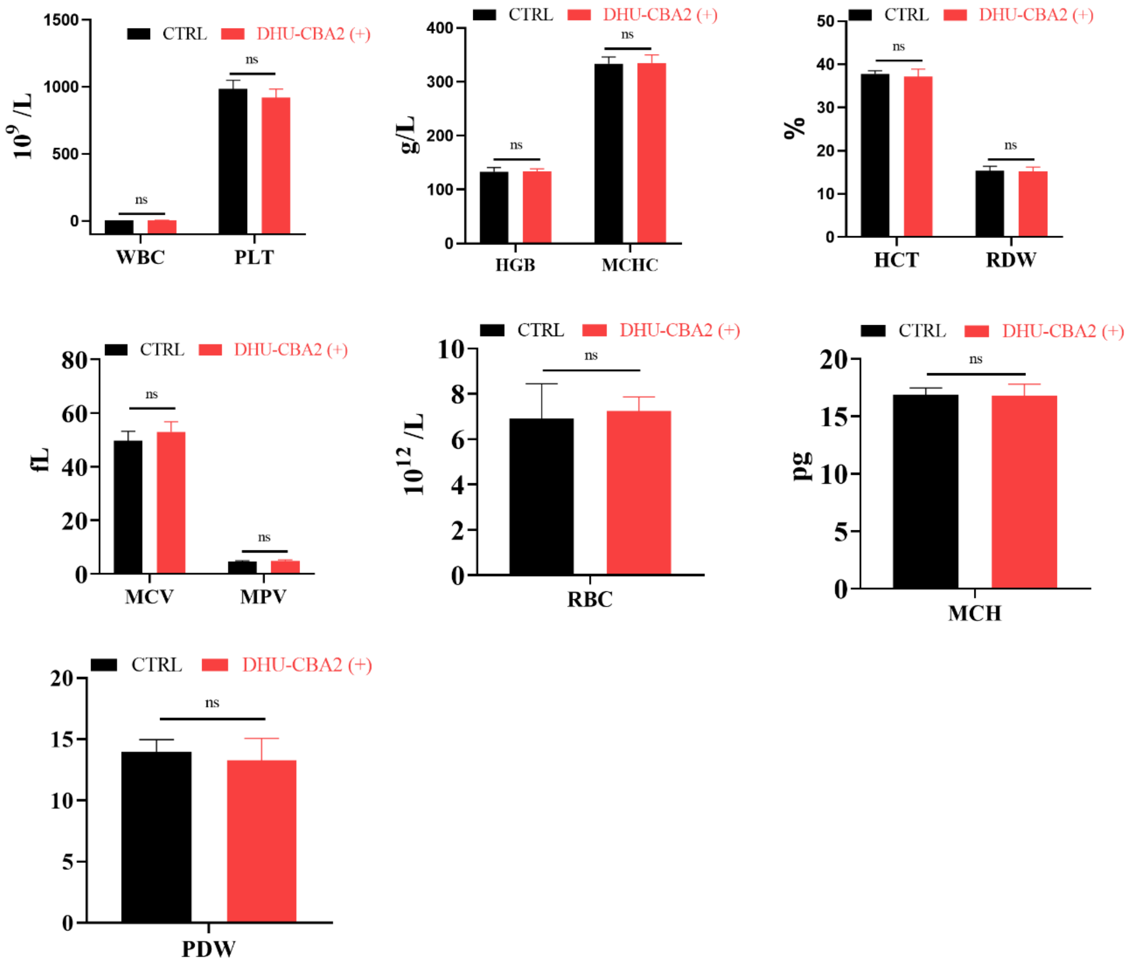

Figure S8. Complete blood count test of subcutaneous LLC-bearing mice after DHU-CBA2 (+) treatment. (+) represents laser irradiation. All data were presented as mean ± SD. Statistical significance was calculated via two-sided Student's *t*-test. ns represents no significant difference.

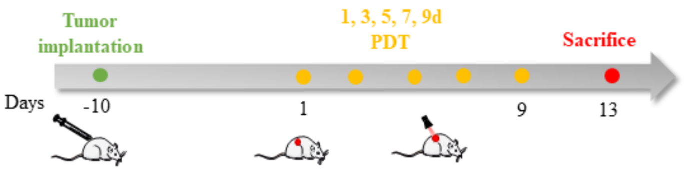

Figure S9. Experimental schedule of tumor treatment in lung cancer spinal metastasis (LC-SM) mice.

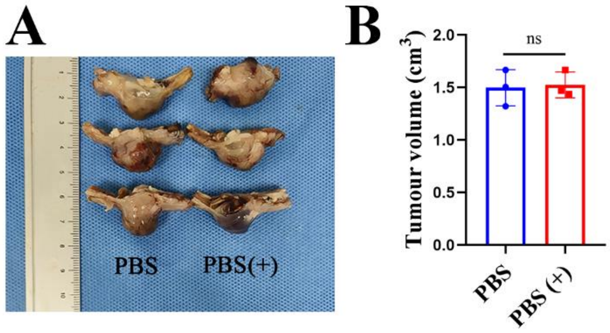

Figure S10. Photograph (A) and tumor volume (B) of lung cancer spinal metastasis (LC-SM) by various treatments at the end of the observation period. "(+)" represents laser irradiation). All data in (B) were presented as mean ± SD. Statistical significance was calculated via two-sided Student's *t*-test. (n=3 individual animals per group, ns represents no significant difference).

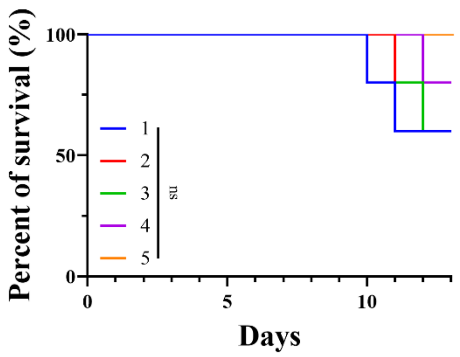

Figure S11. Survival monitoring of LC-SM during the treatment period by various treatments (1: CTRL; 2: DHU-CBA3; 3: DHU-CBA2; 4: DHU-CBA3 (+); 5: DHU-CBA2 (+); "(+)" represents laser irradiation). Statistical significance was calculated via log-rank test. (n=5 individual animals per group, ns represents no significant difference).

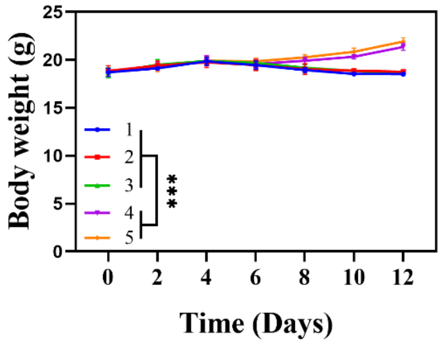

Figure S12. Tumor weight of LC-SM during the treatment period by various treatments (1: CTRL; 2: DHU-CBA3; 3: DHU-CBA2; 4: DHU-CBA3 (+); 5: DHU-CBA2 (+); "(+)" represents laser irradiation). Statistical significance was calculated via two-way ANOVA with Tukey's test. (n=5 individual animals per group, *$p < 0.05$, **$p < 0.01$, ***$p < 0.001$).

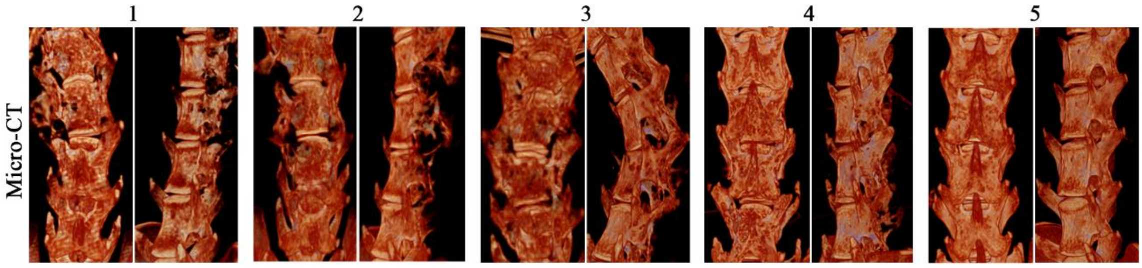

Figure S13. Three-dimensional reconstruction images of spines showing the osteolytic vertebral plate and vertebral column at the end of the observation period (1: CTRL; 2: DHU-CBA3; 3: DHU-CBA2; 4: DHU-CBA3 (+); 5: DHU-CBA2 (+); "(+)" represents laser irradiation).

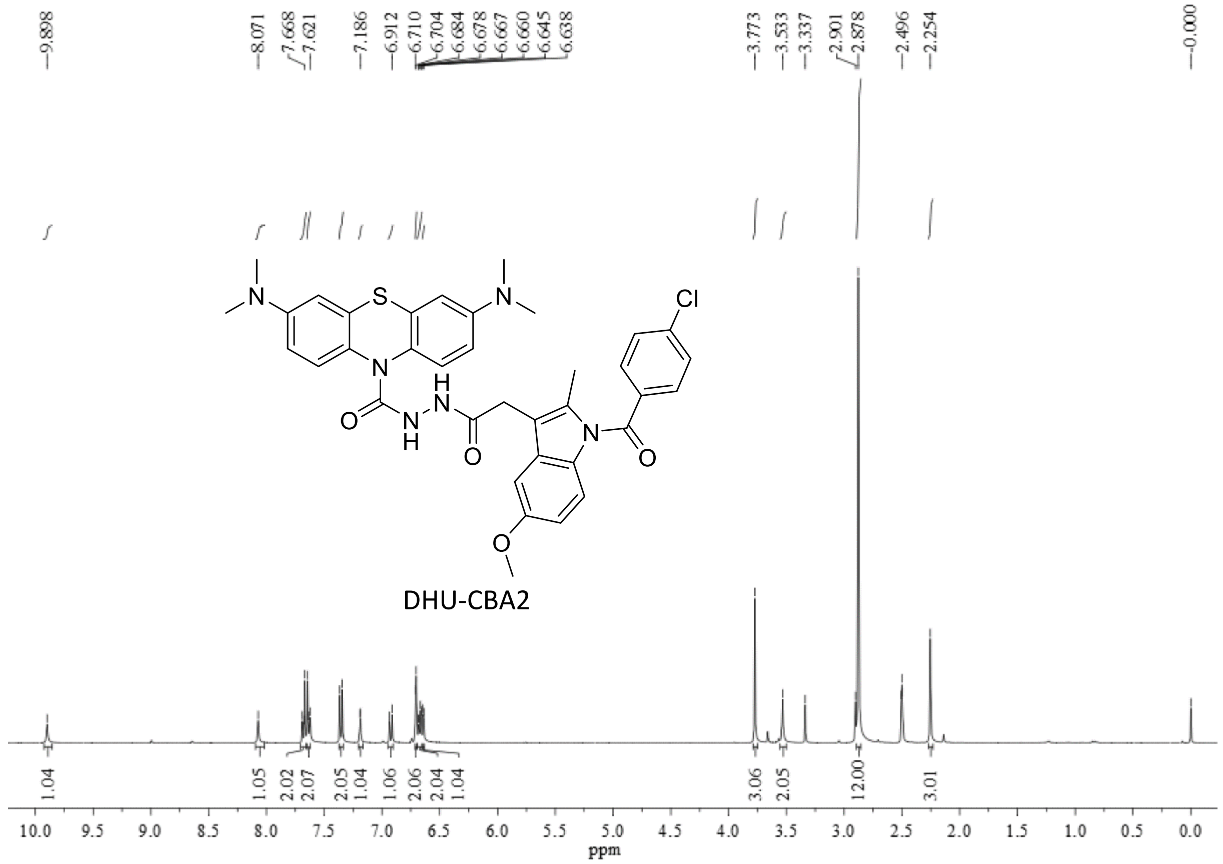

Figure S14. ^{1}H NMR of **DHU-CBA2** in DMSO-d_6.

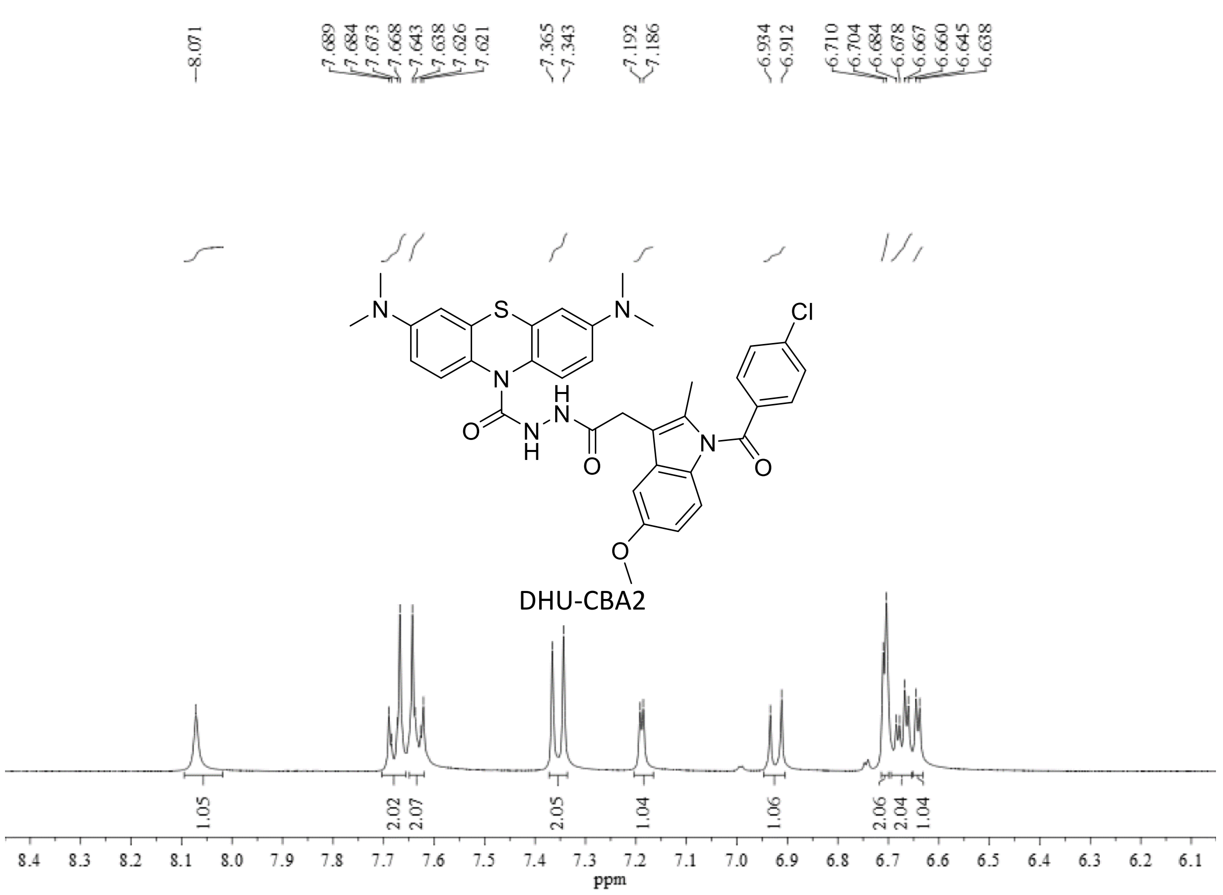

Figure S15. ^{1}H NMR of **DHU-CBA2** in DMSO-d_6.

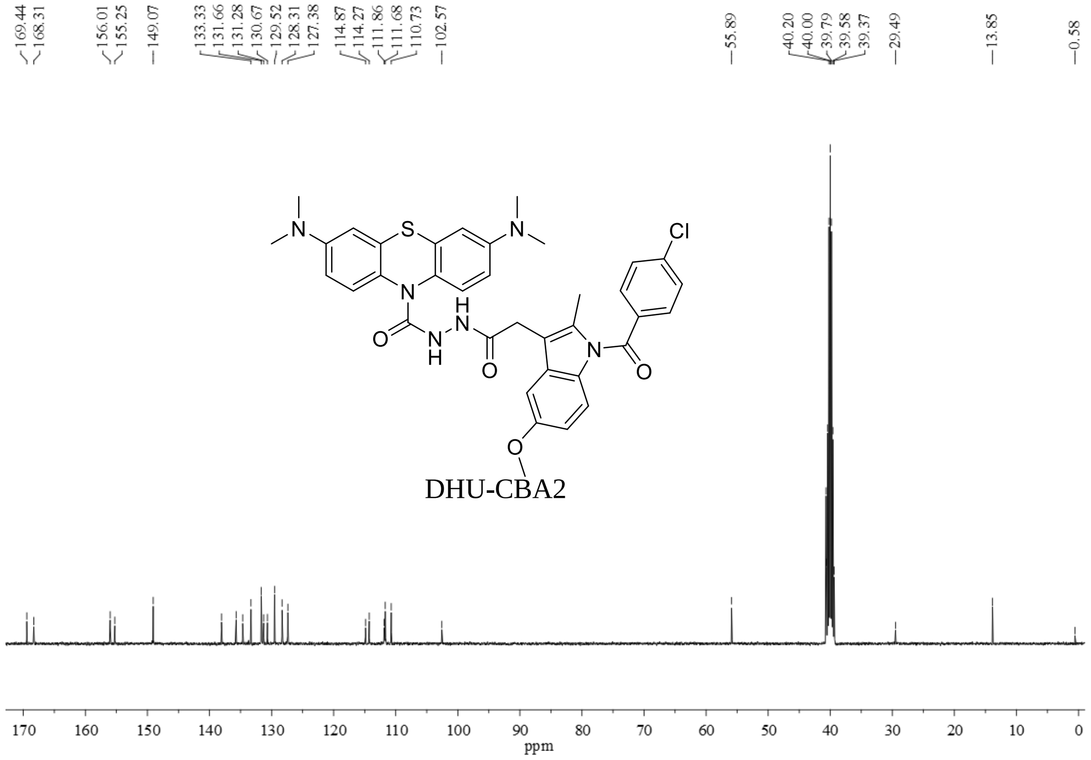

Figure S16. ^{13}C NMR of **DHU-CBA2** in DMSO-d_6.

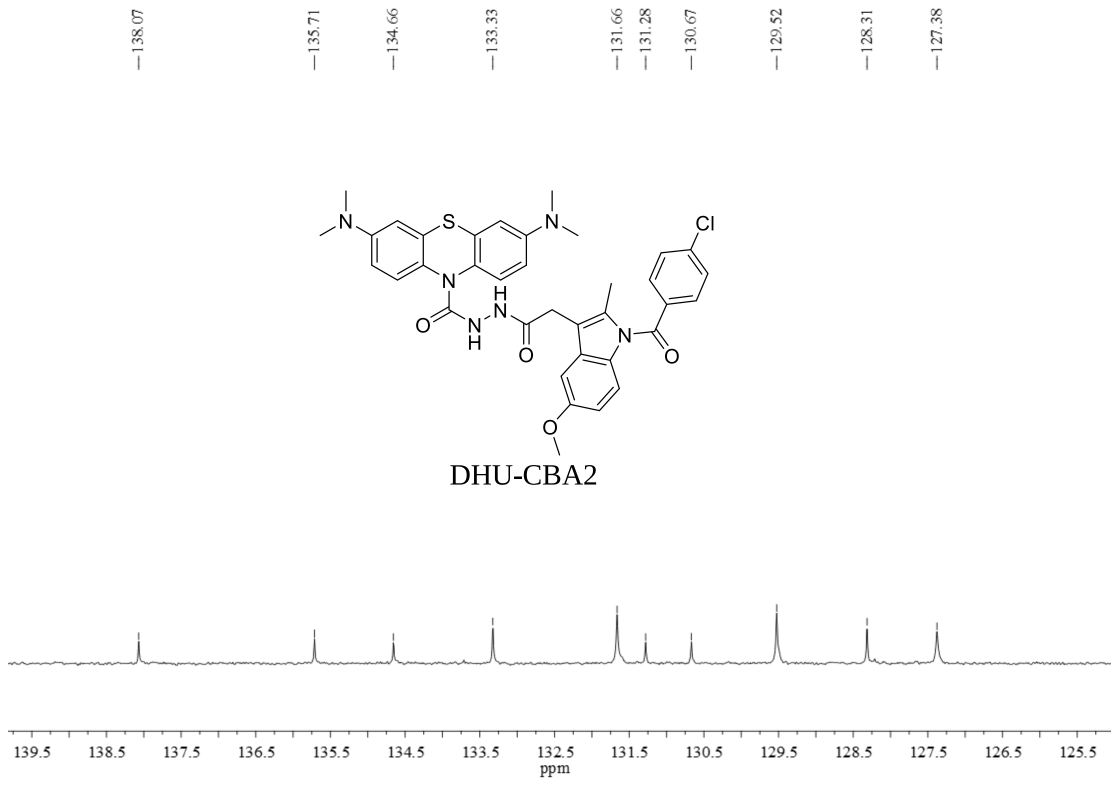

Figure S17. ^{13}C NMR of **DHU-CBA2** in DMSO-d_6.

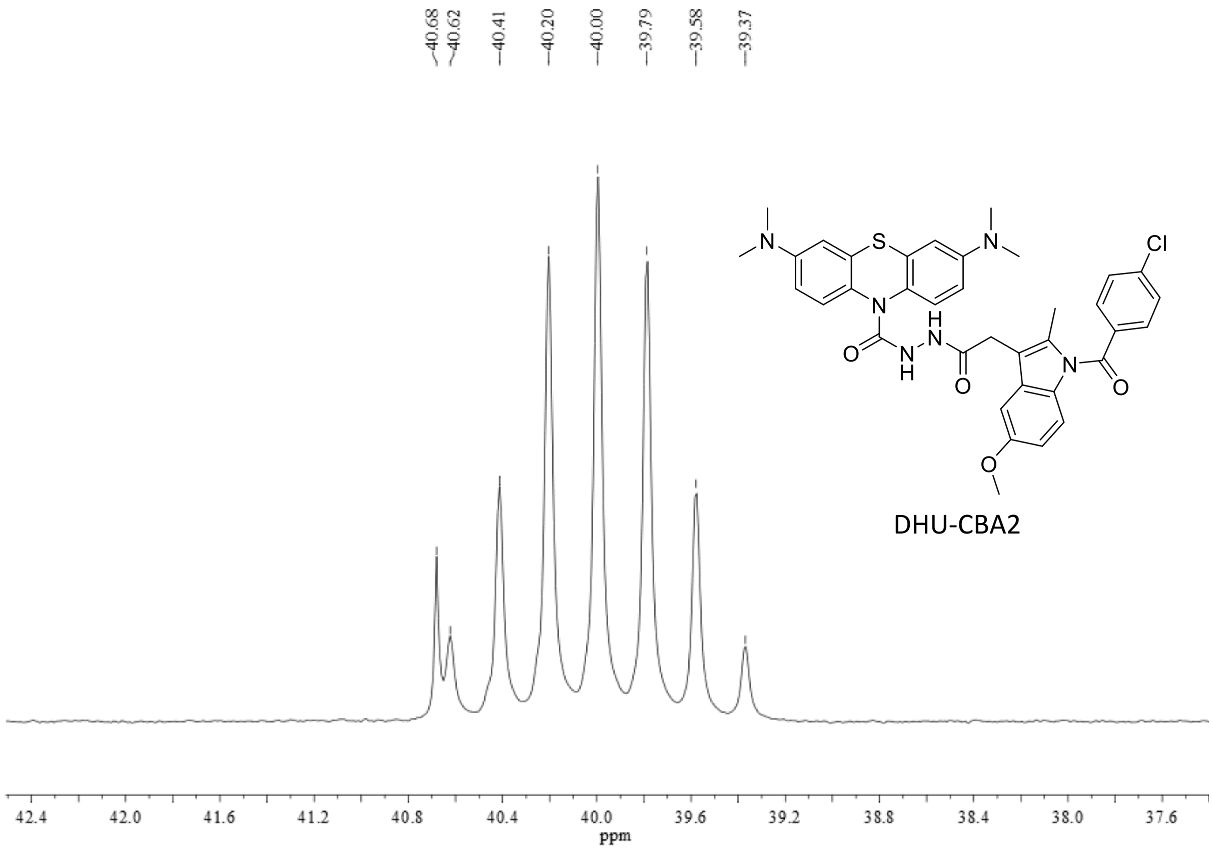

Figure S18. ^{13}C NMR of **DHU-CBA2** in DMSO-d_6.

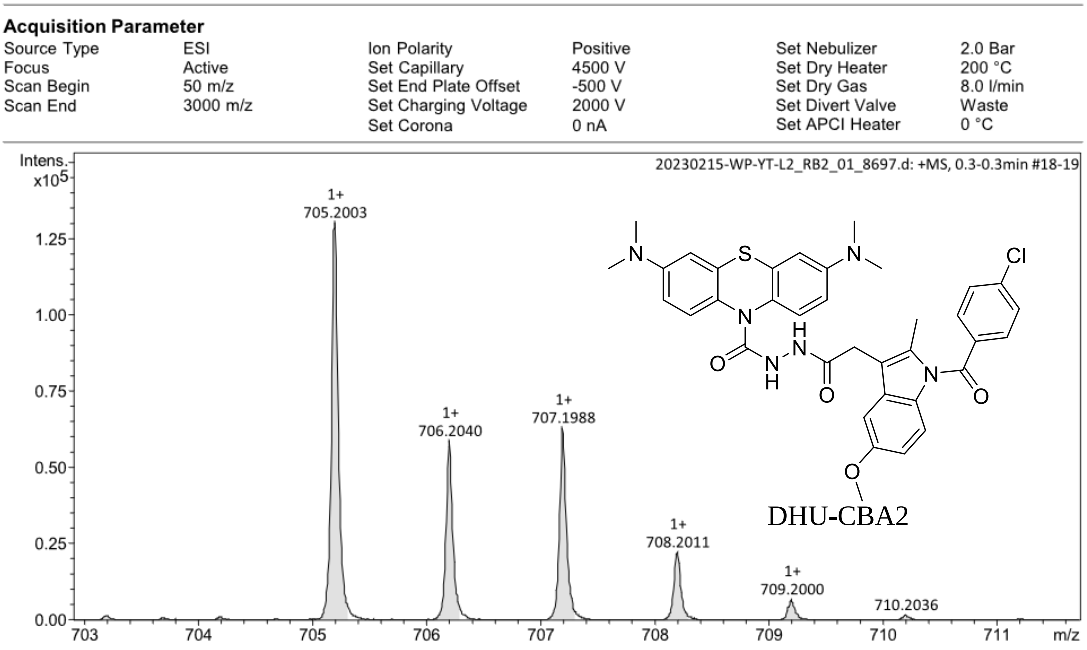

Figure S19. HRMS of **DHU-CBA2**.

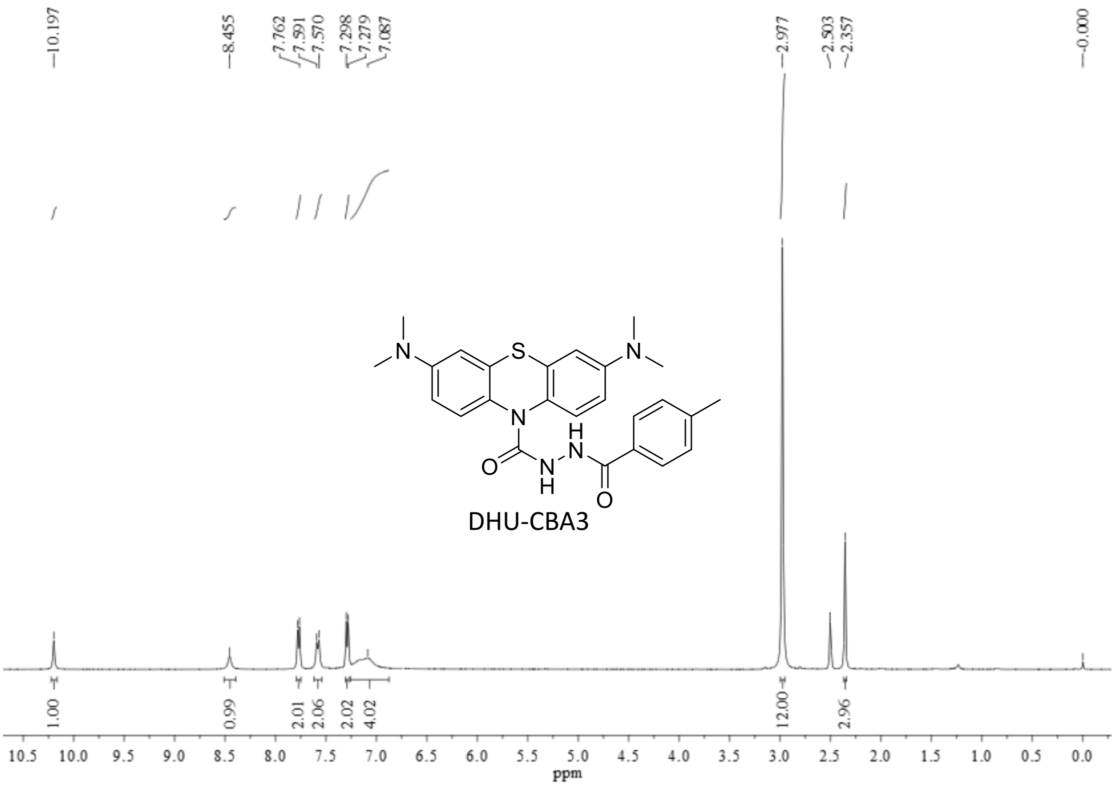

Figure S20. ^{1}H NMR of **DHU-CBA3** in DMSO-d_6.

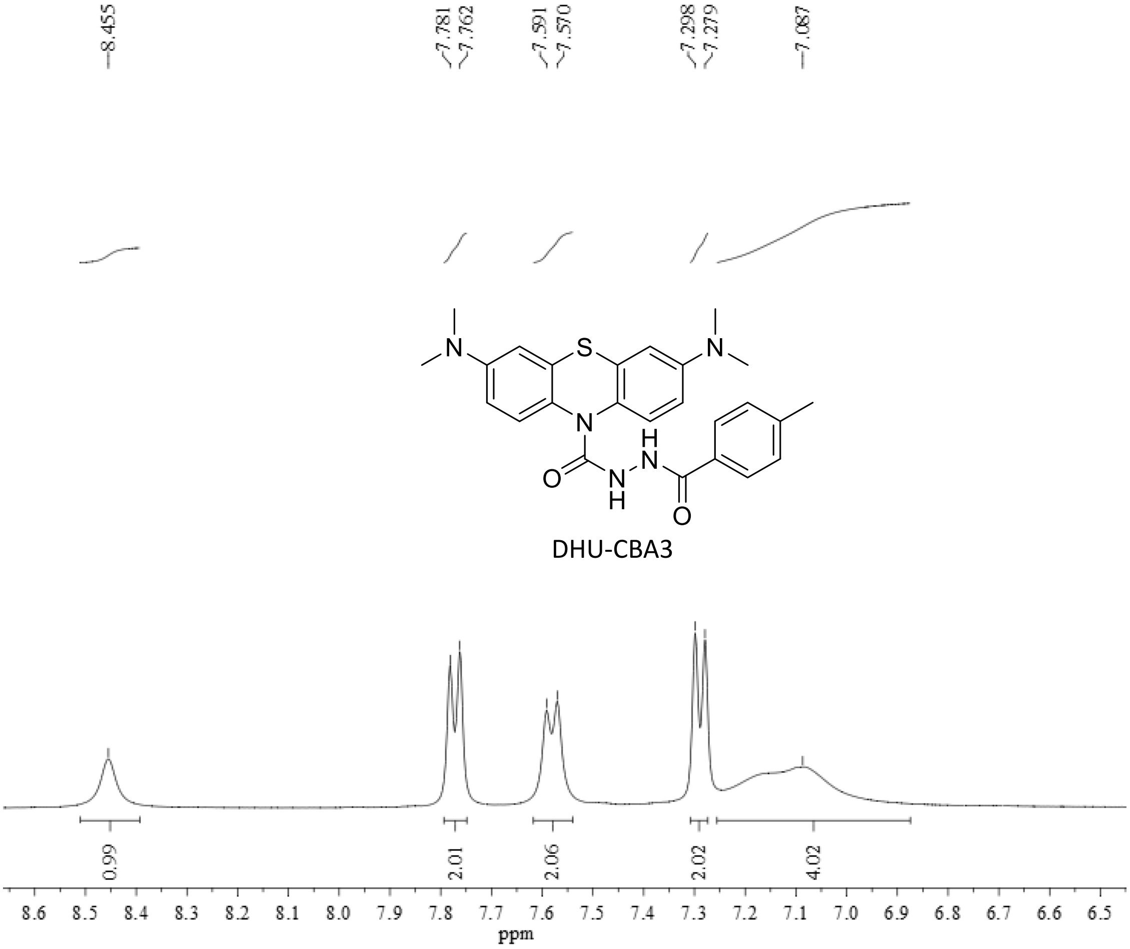

Figure S21. ^{1}H NMR of **DHU-CBA3** in DMSO-d_6.

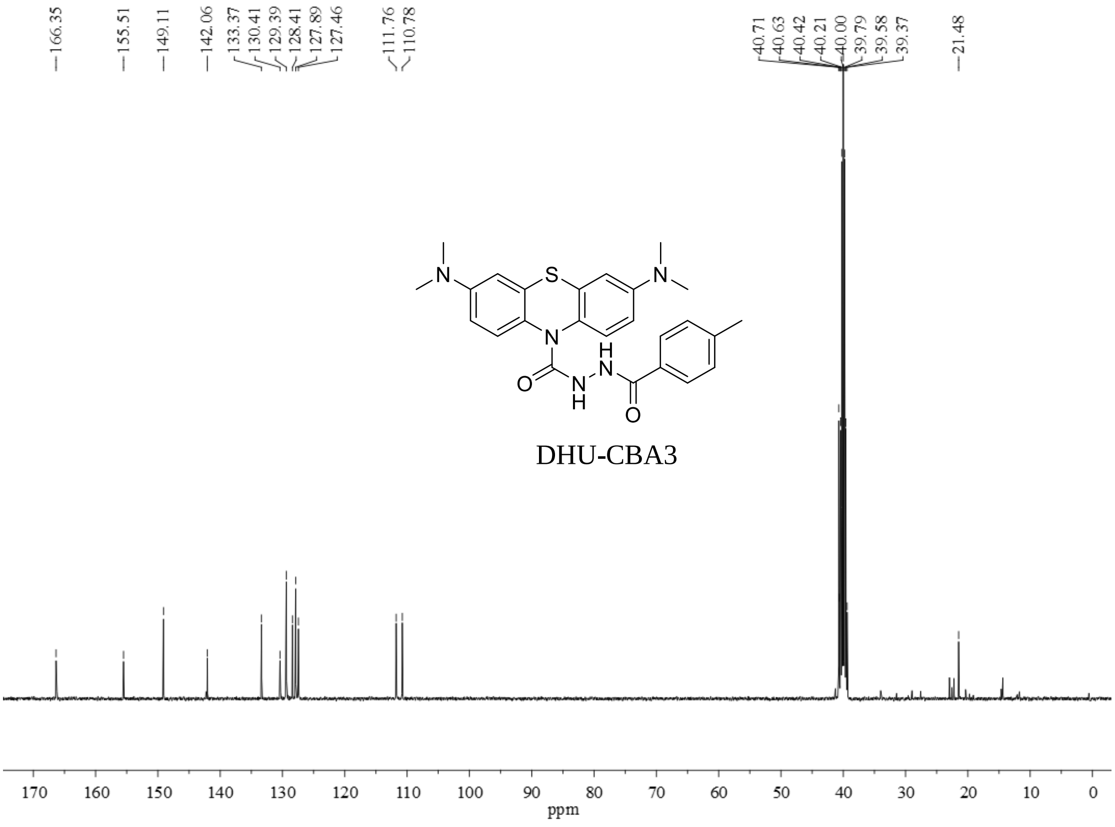

Figure S22. ^{13}C NMR of **DHU-CBA3** in DMSO-d_6.

Acquisition Parameter

Source Type	ESI	Ion Polarity	Positive	Set Nebulizer	2.0 Bar
Focus	Active	Set Capillary	4500 V	Set Dry Heater	200 °C
Scan Begin	50 m/z	Set End Plate Offset	-500 V	Set Dry Gas	8.0 l/min
Scan End	3000 m/z	Set Charging Voltage	2000 V	Set Divert Valve	Waste
		Set Corona	0 nA	Set APCI Heater	0 °C

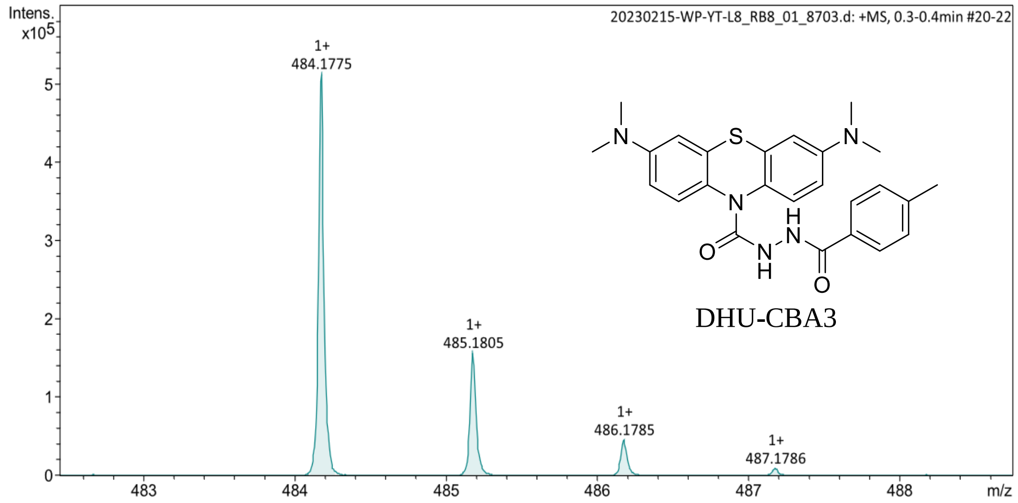

Figure S23. HRMS of **DHU-CBA3**.
